# Echocardiography practice, training and accreditation in the intensive care: document for the World Interactive Network Focused on Critical Ultrasound (WINFOCUS)

**DOI:** 10.1186/1476-7120-6-49

**Published:** 2008-10-06

**Authors:** Susanna Price, Gabriele Via, Erik Sloth, Fabio Guarracino, Raoul Breitkreutz, Emanuele Catena, Daniel Talmor, World Interactive Network Focused On Critical UltraSound ECHO-ICU Group

**Affiliations:** 1 Adult Intensive Care Unit, Royal Brompton Hospital, Sydney Street, SW3 6NP London, UK; 2 1st Department of Anesthesia and Intensive Care, Fondazione IRCCS Policlinico San Matteo, P.zzale Golgi 2, 27100 Pavia, Italy; 3 Department of Anaesthesiology, Skejby Sygehus, Aarhus University Hospital, 8200 Aarhus N, Denmark; 4 Cardiothoracic Anaesthesia and ICU, Azienda Ospedaliera Pisana, via Paradisa 2, 56124 Pisa, Italy; 5 Department of Anesthesiology, Intensive Care, and Pain therapy, Hospital of the Johann-Wolfgang-Goethe University, Theodor Stern Kai 7, 60590 Frankfurt am Main, Germany; 6 Department of Cardiothoracic Anesthesia, Azienda Ospedaliera Niguarda Ca'Granda, P.za Osp. Maggiore 3, 20100, Milan, Italy; 7 Department of Anesthesia, Critical Care and Pain Medicine, Beth Israel Deaconess Medical Center and Harvard Medical School, 330 Brookline Ave., Boston, MA 02215, USA; 8 The WINFOCUS ECHO-ICU Group is a section of the World Interactive Network Focused On Critical UltraSound (WINFOCUS) ( and ). Registered office in via Borgonuovo, Street number 4, Milan, Italy

## Abstract

Echocardiography is increasingly used in the management of the critically ill patient as a non-invasive diagnostic and monitoring tool. Whilst in few countries specialized national training schemes for intensive care unit (ICU) echocardiography have been developed, specific guidelines for ICU physicians wishing to incorporate echocardiography into their clinical practice are lacking. Further, existing echocardiography accreditation does not reflect the requirements of the ICU practitioner. The WINFOCUS (World Interactive Network Focused On Critical UltraSound) ECHO-ICU Group drew up a document aimed at providing guidance to individual physicians, trainers and the relevant societies of the requirements for the development of skills in echocardiography in the ICU setting. The document is based on recommendations published by the Royal College of Radiologists, British Society of Echocardiography, European Association of Echocardiography and American Society of Echocardiography, together with international input from established practitioners of ICU echocardiography. The recommendations contained in this document are concerned with theoretical basis of ultrasonography, the practical aspects of building an ICU-based echocardiography service as well as the key components of standard adult TTE and TEE studies to be performed on the ICU. Specific issues regarding echocardiography in different ICU clinical scenarios are then described.

Obtaining competence in ICU echocardiography may be achieved in different ways – either through completion of an appropriate fellowship/training scheme, or, where not available, via a staged approach designed to train the practitioner to a level at which they can achieve accreditation. Here, peri-resuscitation focused echocardiography represents the entry level – obtainable through established courses followed by mentored practice. Next, a competence-based modular training programme is proposed: theoretical elements delivered through blended-learning and practical elements acquired in parallel through proctored practice. These all linked with existing national/international echocardiography courses. When completed, it is anticipated that the practitioner will have performed the prerequisite number of studies, and achieved the competency to undertake accreditation (leading to Level 2 competence) via a recognized National or European examination and provide the appropriate required evidence of competency (logbook). Thus, even where appropriate fellowships are not available, with support from the relevant echocardiography bodies, training and subsequently accreditation in ICU echocardiography becomes achievable within the existing framework of current critical care and cardiological practice, and is adaptable to each countrie's needs.

## 1.0 Introduction

The application of echocardiography in the critically ill has been well-recognized for several years, principally in patients following cardiac surgery [1-6]. The use of this technique is presently expanding to include diagnosis and monitoring on the general intensive care unit (ICU) [7-14]. Further, as echocardiography is an evolving technology with broadening applications throughout medical and surgical practice, and equipment is becoming cheaper, more portable and more widely available [15-19], it is inevitable and appropriate that medical practitioners other than cardiologists and echocardiographers should seek to develop skills in the performance of ICU echocardiography.

Currently there are no specific guidelines for ICU physicians wishing to incorporate echocardiography into their clinical practice. Despite this, many are recommending that echocardiography should be incorporated into ICU training programs due to its ability to positively impact on patient management [20-35]. However, due to the complexity of issues involved, few have developed specialized national training schemes for ICU echocardiography [28,36,37] and echocardiography accreditation (through EAE, BSE or the ASE) does not reflect the requirements of the ICU practitioner, as they contain heavy emphasis on valvular disease, little haemodynamic monitoring, and an absence of pathology in the critically ill. The World Interactive Network Focused on Critical UltraSound (WINFOCUS, ) is a scientific society committed to the development of high-quality ultrasound in the emergency and ICU setting, including echocardiography. This document has been prepared from recommendations published by the Royal College of Radiologists [38], and British Society of echocardiography [39], European Association of Echocardiography [40] and American Society of Echocardiography [4], together with input from established practitioners of ICU echocardiography to provide guidance to individual physicians (and also to inform program directors, the relevant echocardiographic and intensive care societies, hospital administrators and health care policy makers) of the requirements involved in the development of skills in echocardiography in the ICU setting. Although the authors recognise that ICU clinicians may use ultrasound to image other organ systems, for the purposes of clarity these recommendations are limited to training and accreditation in echocardiography. Recommendations for training of non-radiologists in the imaging of other organ systems exist [38,41], and WINFOCUS projects already include systematic development of a comprehensive ultrasound curriculum for the intensivist and critical care physician [23,42].

## 2.0 Rationale for the use of echocardiography in the intensive care unit

Point-of care echocardiography has become an indispensable tool in the management of the critically ill patient, providing rapid assessment of cardiac function and physiology that complements data available from standard invasive hemodynamic monitoring [8-12,28]. This expanding use of echocardiography may also have been driven by recent publications that have raised concerns regarding pulmonary artery catheterisation [43-48]. Further, the technological advances leading to progressive miniaturization of systems, and advances in echocardiographic techniques (including harmonic imaging, digital acquisition and contrast-enhanced endocardial definition) together with the development of more portable echocardiographic systems has led to their increased use in the ICU. Here, echocardiography is uniquely useful in providing both a monitoring and diagnostic tool for rapid bedside assessment of cardiovascular pathophysiology in the critically ill [12,30,49]. In contrast to standard diagnostic studies, frequently answers to specific questions are required (e.g. loading and volume responsiveness, cardiac output, ventricular function) rather than a fully comprehensive study. Despite enthusiasm for its use by specialists, there is little data to prove the benefit of echocardiography on the ICU. A number of studies have, however, indicated its potential usefulness in changing the diagnosis and management of the critically ill [29-35], together with assessment of ventricular function [10,50-53] fluid responsiveness [54] and the hemodynamics of shock states [8,9,12,55,56].

In addition to standard echocardiography skills, an ICU echocardiographer must be able to interpret findings from both trans-thoracic and trans-esophageal studies, to expeditiously answer specific questions in the context of the rapidly changing pathophysiological status of the critically ill patient, and be accessible for continued echocardiographic monitoring. This, in addition to having the necessary experience to recognize the need for a more detailed study and referral to a more experienced practitioner. Thus, a training and accreditation process targeted at ICU echocardiography must encompass a practice that differs from regular echocardiography, and presents a real challenge. Furthermore, it is essential that ICU echocardiography is incorporated into a co-ordinated echocardiography service, and requires continued communication with cardiologists, echocardiographers and departments of echocardiography.

## 3.0 Levels of competence in intensive care echocardiography

**Figure 1:**
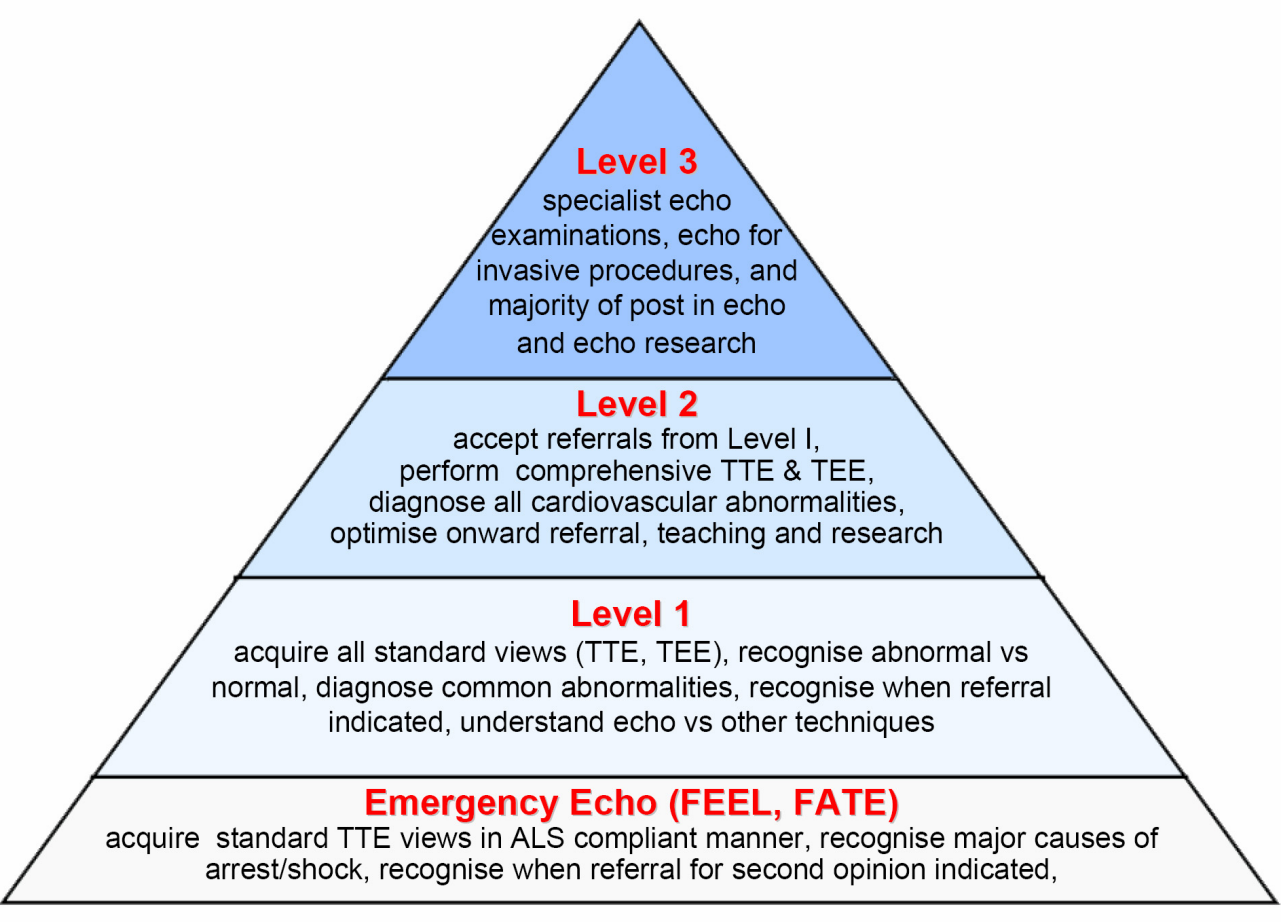
**Proposed Levels of competence of echocardiography in ICU**. According to the widely accepted concept of levels of competence in echocardiography and ultrasound practice (modified from [21]), a 3 Levels structure is proposed. Note that Emergency Echo just represents an entry level, a first very basic step in Echo competence, and does not equate Level 1. TTE = TransThorachic Echocardiography; TEE = TransEsophageal Echocardiography; ALS = Advanced Life Support; FEEL = Focused Echocardiographic Evaluation in Life support; FATE = Focused Assessment with Transthoracic Echocardiography.

Competence in echocardiography and other ultrasound techniques is generally separated into three distinct levels[23,38,57-59] (Figure 1). Although emergency echocardiography is currently being considered as a potential core skill for the acute physician, this covers a limited differential diagnosis, and does not equate to Level I training [30,60,61]. Whilst for emergency physicians this level of competence (peri-resuscitation focused echocardiography) may be regarded as adequate, the practicing ICU physician trained in echocardiography will require competencies beyond this. In order to achieve this, a degree of flexibility, reflecting the practice and time-constraints of the ICU physician, will be required, when compared to achieving an equivalent standard for cardiologists.

### 3.1 Level 1 training

Practice at this level [38] would usually require the following abilities:

• To perform echocardiographic examinations safely and accurately and acquire all standard views

• To recognise and differentiate between normal anatomy/physiology and pathology

• To diagnose common abnormalities within the cardiovascular system

• To recognize when a referral for a second opinion is indicated

• To understand the relationship between echocardiographic imaging and other diagnostic imaging techniques

The Level 1 practitioner is thus not "accredited" in echocardiography, but rather has achieved competency in performing high quality echocardiographic examinations specifically targeted to the ICU patient population (for example, peri-resuscitation echocardiography), with clear knowledge of their limitations and requirement for referral where abnormalities are found. This level of practice is commonplace in the ICU, and is analogous to interpretation of chest radiography or electrocardiography; here, where an abnormality is seen but the diagnosis not obvious, the practitioner routinely seeks more expert advice.

While training programs vary internationally, this level of competence may be gained during conventional postgraduate specialist training programs in ICU. Alternatively, where such training is not available as part of a critical care fellowship it may be acquired during a specialized training course followed by guided practical experience in the ICU. This supervised practical experience could be combined with on-line learning (blended learning) to provide a structure to the training programme.

### 3.2 Level 2 training

Practice at this level [38] would usually require the following abilities:

• To accept and manage referrals from Level 1 practitioners

• To perform echocardiographic examinations (TEE and TTE) safely and accurately and acquire all standard views

• To recognise and correctly diagnose almost all conditions within the cardiovascular system

• To have sufficient understanding of echocardiographic depiction of pathology to optimize the referral of the patient if the condition falls outside the practitioner's skills

• To perform echocardiography for common non-complex echo-guided invasive procedures (i.e. pericardiocentesis)

• To teach echocardiography to trainees and Level 1 practitioners

• To conduct some research in echocardiography

The training required for this level of practice would usually be gained during a period of subspecialty training which may either be within or after the completion of a specialist ICU training program, and would require interpretation of both TTE and TEE. This would equate to the level of training in radiology (or echocardiography for an imaging cardiologist) at the time of completion of specialist training, or echocardiography accreditation (i.e. BSE/ASE/EuroEcho).

### 3.3 Level 3 training

Accreditation (at Level 2) was devised initially in echocardiography to set the minimum standard for echo technicians and has since been adopted by clinicians to show a basic level of competency. It is important, however, to remind practitioners that this is a baseline minimal standard of competency. Level 3 is an advanced level of practice[38], which includes some or all of the following abilities:

• To accept tertiary referrals from Level 1 and Level 2 practitioners

• To perform specialised echocardiographic examinations

• To perform advanced echo-guided invasive procedures

• To conduct substantial research in echocardiography

• To teach echocardiography at all levels

• To be aware of and to pursue developments in echocardiography

This would equate to a consultant cardiologist/intensivist with a subspecialty practice which includes a significant commitment to echocardiography in the ICU.

The boundaries between these three levels are difficult to define precisely, and the above are a guide to different levels of competence and experience. In the detailed syllabus attached to this document in Appendix 4 an attempt is made to indicate more specifically the type of experience required for each level of training.

## 4.0 Intensivist vs. Cardiologist

Despite echocardiography having historically been exclusively the domain of the cardiologist, with the development of TEE and its use in the cardiac surgical setting, its use has extended to anaesthetists. Indeed, in the UK > 90% of TEE studies are now undertaken by anaesthetists. This extension of echocardiography beyond the specialty of cardiology is likely to continue. Focused echocardiography as an adjunct in the peri-arrest period is likely to become a core competency for acute medicine trainees [61], and with the increasing recognition of the potential role of echocardiography on the ICU, international training recommendations now include basic echocardiography [62]. Whether this should extend to intensivists becoming fully trained and accredited in echocardiography remains, for some, contentious.

Although specialist training in cardiology will generally include TTE to level 2 equivalence (or accreditation), cardiology training programmes often excludes formal exposure to general ICU. Further, the specific conditions most relevant to the ICU (i.e. sepsis, filling status, ventricular function related to the level of inotropic support, heart-lung interactions in mechanically ventilated patients) are not generally addressed, and the use of echocardiography as a monitoring tool is not taught. In addition, the diagnosis of pathology in the ICU is on occasion better resolved with TEE rather than TTE. Finally, ready availability of appropriately trained consultant cardiologist-echocardiographers for the repeated examinations required in the ICU is not always possible.

By contrast, the practicing intensivist has a wide knowledge of the pathophysiology of the critically ill, and is readily available, but may not have the necessary echocardiographic skills. When such intensivists have been appropriately trained to perform TTE, TEE and physiological echocardiography, they will be ideally positioned to perform ICU echocardiography. Development of an appropriate program will require extensive cooperation and support form a hospital's cardiology service. This support extends beyond initial training to include support for quality assurance, maintenance of competency among the ICU practitioners and most importantly backup in the case of a difficult diagnosis or one beyond the daily scope of practice of the ICU echocardiographer. An example of such a diagnosis could be evaluation of a mitral valve for suitability for repair.

## 5.0 Scope of knowledge for an ICU echocardiography practitioner

These recommendations are concerned with theoretical basis of ultrasonography, the practical aspects of building an ICU-based echocardiography service as well as the key components of standard adult TTE and TEE studies to be performed on the ICU [39]. These recommendations are summarized in Appendices 1 through 3. The intended benefits of these recommendations are to support the development of local protocols and quality control programs for echocardiography on the ICU, promote quality by defining a minimum dataset of descriptive terms and measurements, and a systematic approach to constructing a report, to facilitate the accurate comparison of serial echocardiograms performed in ICU patients. These recommendations are in line with recommendations from the European Association of Echocardiography [63,64] and the British Society of Echocardiography [65].

### 5.1 Principles of ultrasonography and echocardiography

Without a solid understanding of the physical basis of ultrasonography the practitioner will not be able to perform and interpret an exam accurately. Thus, any programme should include training in the appropriate selection of ultrasound modality and in the mechanics of setting up and running an intensive care echocardiography service. A syllabus for theoretical training is presented in Appendix 1.

### 5.2 Trans-thoracic echocardiography (TTE)

Recommendations for the minimum image and analysis dataset comprising a standard, comprehensive adult TTE are shown in Appendix 2. Although there is broad agreement over what views and recordings are essential in any comprehensive study [39], in contrast to standard, out-patient diagnostic echocardiography however, focused studies may be appropriate (i.e. to exclude pericardial collection, or to monitor pulmonary arterial pressures in response to therapeutic intervention). These should be identified as being focused studies, and are not covered by these minimum standards, although relevant parts of the recommendations can be utilized. When the condition or acoustic windows of the patient prevent the acquisition of one or more components of the Minimum Dataset, or when measurements result in misleading information (e.g. off-axis measurements) this should be stated.

### 5.3 Trans-esophageal echocardiography (TEE)

Recommendations for the minimum image and analysis dataset comprising a standard, comprehensive adult TEE are shown in Appendix 3. Althought there is broad agreement over what views and recordings are essential in any comprehensive study [4,40], however, as with TTE, on the ICU focused studies may be appropriate on occasion and these should be clearly identified as such.

### 5.4 Clinical practice of echocardiography in the ICU

There are conditions that occur in the ICU that require addressing specifically when considering ICU echocardiographic training. Depending on the experience of the practitioner and the clinical status of the patient, in general once a standard study has been performed, these may then be the subject of focused/detailed examinations, particularly where a change in therapy has been instituted, where echocardiography may be used to monitor the patient response. These include:

• Peri-resuscitation echocardiography

• Systolic function and regional wall motion abnormalities

• Diastolic function

• Hypovolemia and volume responsiveness

• Tamponade and pericardial disease

• The sepsis syndromes

• Effects of pre-load and afterload and assessment of filling status

• Acute cor pulmonale

• Hypoxemia

• Complications of acute MI

• Chest trauma

• Assessment of shock

• Failure to wean from mechanical ventilation

• Hemodynamic measurements

The specific details regarding echocardiography for each of these clinical scenarios are addressed in Appendix 5.

## 6.0 Educating the ICU echocardiography practitioner

Accrediting authorities in echocardiography are not generally proscriptive in how the trainee achieves the standard required for accreditation, but rather in setting the standard [BSE, EAE, ASE]. Education of cardiologists in echocardiography is generally achieved as part of a standard training scheme with supervised, hands-on experience combined with attendance at existing national and international courses. Similarly, cardiothoracic anaesthetists wishing to become accredited in TEE will generally spend time in a cardiothoracic centre undertaking a specific fellowship, together with attendance at appropriate courses – some directed specifically towards examination preparation [59]. The main challenge for trainees and existing consultant intensivists is how to achieve training and accreditation in ICU echocardiography when specific fellowships and/or diplomas are not available. Time limitations and lack of access to training in the ICU generally causes an issue for intensivists – together with lack of expertise in this rapidly expanding field. Hence, this document seeks to suggest ways in which this training may be achieved, whilst maintaing the standards of accreditation required by the echocardiography societies.

### 6.1 Theoretical Training

Training in echocardiography encompasses both theoretical and practical elements. Theoretical background is prerequisite for the performance and interpretation of an echocardiographic exam [66] (Accessed August 2008). The required elements of theoretical training are outlined in Appendix 1. This element of training may be best delivered by linking with courses run by university departments accredited by the Consortium for the Accreditation of Sonographic Education (CASE) – or other basic ultrasound/echo courses.

### 6.2 Practical training

A syllabus for ICU echocardiography structured into three levels of training is proposed, incorporating theoretical training on anatomy and pathology and a practical syllabus listing conditions which should be included in the experience of the trainee. The levels and potential modes of training are shown in Appendix 4. Practical experience should be gained under the guidance of a named supervisor trained in echocardiography within a department. This would normally be a department of echocardiography and/or in the ICU, however, there may be some areas of echocardiographic practice which are may be better covered in other departments, such as intra-operative echocardiography for TEE.

A major challenge for the ICU clinician is access to appropriate echocardiographic training. Where a fellowship dedicated to ICU echocardiography is not possible, under close supervision, echocardiography is well-suited to blended learning. Thus, over a period of training the practitioner would progress from performance of peri-resuscitation echocardiography to be competent at performance and interpretation of all components of a comprehensive study, as well as focused echocardiography in specific clinical scenarios. Throughout this training a competency assessment sheet should be completed with each element signed by the supervisor. This would determine in which areas(s) the trainee may wish to practise independently (for example, assessment of filling status) whilst undergoing training in other areas. It is important to note, however, that accreditation in ICU echocardiography should be to the same standard as other types of echocardiography – completion of any one or all of the modules would not, therefore, equate to accreditation. The principles and requirements for the different levels of training are as follows:

#### Level 1

• A modular approach to acquiring echocardiographic skills (Appendix 4) under supervision (blended learning)

• As different trainees acquire skills at different rates, the end-point of each part of the training programme will be judged by an assessment of practical competence i.e. competency-based assessment

• Examinations should encompass the full range of pathological conditions listed in the syllabuses

• A logbook listing the number and type of examinations undertaken by the trainee themselves should be kept

• An illustrated logbook of specific normal and abnormal findings may be appropriate

• Training should usually be supervised by at least a Level 2 practitioner

#### Level 2 (leading to accreditation)

• This would usually require at least 1 year of experience at Level 1, with the equivalent of at least one session per week

• A significant further number of examinations should have been undertaken in order to encompass the full range of conditions and procedures encountered in each module

• A logbook listing the numbers and types of examinations undertaken by the trainee should be maintained

• An illustrated logbook of specific normal and abnormal findings will be presented

• Supervision of training should be undertaken by someone who has achieved at least Level 2 competence and has had at least 2 years' experience at that Level

• On completion of Level 2 training, the ICU echocardiographer would normally be expected to obtain ICU echocardiography accreditation (section 6.3.2)

#### Level 3

• This would require practitioners to spend a significant part of their time undertaking echocardiographic examinations, teaching, research and development

• They would have spent a continuous period of sub-specialist training in which ICU echocardiography will have been a significant component

• They would be able to perform specialised examinations at the leading edge of echocardiographic practice such as the use of intravascular ultrasound contrast agents and the performance of advanced ultrasound-guided invasive procedures

### 6.3 Documentation of training

#### 6.3.1 Level 1 training – Logbook

The log-book should be composed of:

• a set of copies of signed reports enclosed in a folder or binder

• all reports submitted will carry the signature of the trainee & supervisor

• the main sections will include studies performed:

- Peri cardiac arrest

- Focused studies for haemodynamic measurement

- Focused studies for specific diagnosis

- Comprehensive diagnostic studies

• the studies should include focused and comprehensive assessment of more than one example of each of the following:

- Tamponade

- Cardiogenic shock

- Hypovolemia and Volume responsiveness

- Chest trauma

- Pulmonary embolism

- Septic shock

- Acute pulmonary edema

#### 6.3.2 Level 2 training – Accreditation

Accreditation in ICU echocardiography should be under the auspices of the appropriate regulatory national and international bodies, and must not represent a substandard or simplified accreditation. Indeed, many would argue that due to the rapidly changing pathophysiology of the critically ill patient, ICU accreditation (with its requirement for both TTE and TEE, together with in depth haemodynamic assessment) should be regarded an upgrade or extension of existing accreditation. In practice, the most appropriate process would be for the existing accrediting authorities (ASE, BSE, EAE) to modify existing examination and logbook content to reflect the clincal situation and casemix of the critically ill. Indeed, similar collaboration between ACTA and the EAE, and ACT and the BSE resulted in TEE accreditation being widely available.

Not all practitioners will wish to or need to undergo echo accreditation, however, where required, accreditation must be equivalent to existing echocardiography accreditation programmes, and ideally be run under the direction of the appropriate organisations. Thus:

• The accreditation process will be run as a regulatory certificate of competence

• In each country, the accreditation should be developed in accordance with national cardiological, echocardiographic and intensive care societies (analogous to the development of TEE accreditation)

• The accreditation process is designed to teach and test competence at performing, interpreting and reporting studies to the appropriate level. For TEE studies, probe insertion will not be tested, however, interpretation of TTE and TEE images will be required as the two procedures are complementary on the ICU

• Each candidate for accreditation must enroll with a suitably qualified supervisor who undertakes to train and supervise (Appendix 3) and to arrange visits to other centres if there are difficulties obtaining an adequate case-mix locally

• The accreditation process should require that the candidate submit a log-book and pass a written examination

• Applications for approval as supervisor will be invited. Either one or two supervisors at each centre will be approved based on demonstration of competence at echocardiography and evidence of continuing practice

• Accreditation is a minimum standard and cannot be regarded as a guarantee of continuing competence. Successful candidates will be expected to begin a process of continuing medical education towards re-accreditation (minimum of 50 studies per annum, ideally in excess of 100).

• The re-accreditation process will include evidence of continuing clinical activity, distance learning and attendance at courses and conferences.

##### 6.3.2.1 Level 2 training – Logbook

The log-book will be collected over a period of up to 24 months, as part of a recognised ICU echocardiography accreditation programme (i.e. BSE). Where appropriate, studies performed during Level 1 training may be used. In order to allow flexibility for applicants without access to both modalities of echocardiography, accreditation will be based on interpretation of TTE *and *TEE (examination) however, the logbook may contain studies of either TTE, TEE or TTE & TEE, depending on the practice of the applicant. In addition, where the applicant already holds current accreditation in either TEE or TTE, the number of required studies will be reduced accordingly (Table 1).

**Table 1:** Number of cases required for logbook depending upon accreditation already held by applicant.

**Accreditation already held**	**Access to TTE and TEE**	**Access only to TTE**	**Access only to TEE**
**Standard TTE**	125 (min 50 TTE + 50 TEE)	125 TTE	125 TEE

**Standard TEE**	125 (min 50 TTE + 50 TEE)	125 TTE	125 TEE

**ICU TTE**	75 (min 50 TEE)	N/A	N/A

**ICU TEE**	75 (min 50 TTE)	N/A	N/A

**Nil**	250 (min 100 TTE + 100 TEE)	250 TTE	250 TEE

TTE = trans-thoracic echocardiography; TEE = trans-oesophageal echocardiography; Nil = no accreditation held; min = minimum number required; Access = availability/access of echocardiographic modality and training to the applicant in training. N/A = not applicable

##### Notes

• The log-book will be a set of copies of signed reports enclosed in a folder or binder

• All reports submitted must carry the signature of the candidate.

• Where intra-operative TEE studies are performed before and after bypass during the same operation, these count as one study. A study performed for the same patient on another occasion counts as a separate study.

• The studies should include comprehensive assessment of more than one example of each of the following:

- Tamponade

- Cardiogenic shock

- Hypovolemia and assessment of volume responsiveness

- Chest trauma

- Pulmonary embolism

- Septic shock

- Acute pulmonary edema

• A letter from the supervisor must be submitted with the completed log-book certifying that the studies have been recorded by the candidate.

### 6.4 Maintaining competence

• The number of exams required to maintain competency is more than 50 and preferably more than 100 examinations per annum [40,59]

• CME/CPD should be undertaken which incorporates elements of ICU echocardiography practice

• Regular audit of the individual's echocardiographic practice should be undertaken to demonstrate that the indications, performance and diagnostic quality of the service are all satisfactory

## 7.0 The practice of echocardiography in the ICU

ICU echocardiography should be set up as part of the hospital's comprehensive service. Thus, standards of an accredited centre will apply with respect to staff, organisation and equipment [64], to guarantee standards of performing and reporting examinations, even when focused studies are being performed. Thus, even when performing a TOE on a sedated, intubated and ventilated patient, the operator should not be responsible for anaesthesia, the airway or the haemodynamics of the patient [40,63]. This is particularly relevant in the haemodynamically unstable patient, and/or where more advanced studies (i.e. pacing optimisation/stress echocardiography) are performed.

### 7.1 Medicolegal Aspects of ICU echocardiography

#### 7.1.2 Consent

Critical care is delivered on the principle of respect for patients' dignity and cultural backgrounds. Invasive procedures should be fully explained to the patient and undertaken only after consent is obtained. An example of such a procedure is TEE. Exceptions are usually allowed in the case of life threatening emergency and/or where the patient is unable to give informed consent, but this may vary between countries. Where a patient is able to give consent, development of procedure specific consent (PSC) and patient/relative information leaflets (particularly for TEE) may be useful. Where possible, consent should be obtained before images are used by the practitioner for training other practitioners in accordance with local guidelines. Where research is undertaken, ethical approval must be obtained, and consent (for acquisition and use of both TTE and TEE images) sought according to the research protocol agreed and local guidelines.

### 7.2 Patient safety

With respect to undertaking TTE, the practitioner will obtain training in the following aspects of patient safety:

• Indications and potential pitfalls in interpretation of TTE

• Limitations of some minaturised devices with basic configuration [15]

• Infection control

- Knowledge of local and national guidelines regarding infection control

• Electrical hazards

• Care of the probe

• Probe manipulation

With respect to undertaking TEE, the practitioner will obtain training in the following aspects of patient safety:

• Potential complications and their avoidance

- Indications, relative and absolute contra-indications for TEE

- Anatomy and physiology of the esophagus

- Diseases of the esophagus

- Endoscopy skills – observation of 50 intubations and > 50 successful supervised intubations

- Contraindications of performing procedure – absolute and relative

- Patient assessment before, during and post-procedure

- Knowledge and understanding of the pharmacology of drugs used in TEE

- Knowledge and understanding of the process of obtaining informed consent

• Informed consent – see section 6.1

• Infection control

- Knowledge of local and national guidelines regarding infection control

- Knowledge of local and national guidelines regarding probe sterilisation, cleaning and tracking

• Electrical hazards

• Care of the probe

- Procedures for checking integrity of the probe prior to each use

- Infection control (see above)

• Probe manipulation[4]

### 7.3 Storage of exams

The permanent recording of images, where appropriate, is essential for the purposes of correlative imaging, future comparison and audit. A study should be performed and stored on video/digital format to be available for review and comparison. Digital storage is preferred, as it more easily facilitates serial comparison of studies which is invaluable in the management of the critically ill [63].

### 7.4 Reporting and documenting examinations

A system for recording the results of any echocardiographic examination in the patients' record is mandatory [63]. Content of written reports should be as far as possible standardized to improve clarity and encourage proper nomenclature and should include important haemodynamic data and the level of cardiorespiratory support at the time of the examination. [63] Responsibility for generating a timely report is shared between the trainee and his/her supervisors.

### 7.5 Continuous quality improvement

A system for reviewing echocardiographic examinations and their reports within the ICU and in conjunction with the echocardiography department of the hospital is important. Regular audit of the number, quality of studies and their reports should be performed. Responsibility for continuous quality improvement is shared between the trainee and his/her supervisors.

### 7.6 Echocardiography research in the ICU

An expectation of level 2 and 3 practice is the performance of research in ICU echocardiography. As with all research in the ICU setting, obtaining consent to participate in research is challenging. Whether undertaking research using TTE or TEE, ethical approval and consent must be obtained according to local and/or national guidelines.

## 8. Conclusion

To a large extent, intensive care has developed in parallel, but separate from cardiology, with little use of the range of physiological measurements and assessments that echocardiography has to offer. This is unsurprising, given the different training pathways that have been followed by intensivists and cardiologists. Indeed, echocardiography has been regarded as the province of the cardiologist since its inception nearly 50 years ago. With increased sub-specialization in the cardiological field, and the development of acute cardiac care as a specific sub-specialty, it is likely that some cardiologists of the future will extend their role into the cardiac ICU setting.

The intensivist is, however, uniquely positioned to understand and balance the multi-system pathophysiological variables that underpin critical illness – both in the cardiac and general ICU setting. It is entirely appropriate therefore that intensivists should be able to develop echocardiographic skills, but consequently it is crucial that they should be able to obtain the necessary echocardiographic training such that they are able to practice this physiological investigation to a high level within their intensive care units. Not all intensivists will wish to (or need to) obtain this higher level training in echocardiography, however, every intensivist undergoing training should be competent in peri-resuscitation echocardiography. Where intensivists do wish to develop their echocardiographic skills, their goal should be a recognised accreditation through an established national or international body. This is achievable through collaboration between the existing accrediting authorities and the relevant national and international intensive care socieities – in a manner analogous to that in which TEE accreditation for anaesthetists was developed. The process by which training is obtained remains a challenge, however, the combination of practical training under supervision with interactive on-line learning will facilitate this process where there is no opportunity for an ICU echocardiography fellowship. As with TEE training for cardiac anaesthetists, it is important that this accreditation should be regarded as an entry level requirement for those who wish to develop echocardiographic skills relevant to the ICU setting. ICU echocardiography should not be practiced in isolation, but in conjunction with consultant (echo)cardiologists as part of a comprehensive echocardiography service. In this way, one would hope to see the performance of high quality, relevant echocardiography, together with well-devised and executed research in this rapidly expanding field.

## Competing interests

With regard to the manuscript "Echocardiography practice, training and accreditation in the intensive care: strawman document for the World Interactive Network Focused on Critical Ultrasound (WINFOCUS)", the authors declare not to have any financial or non financial competing interest.

## Authors' contributions

SP conceived and wrote the substantial part of the manuscript and of the Appendices. GV gave a major contribution in writing manuscript and the Appendices. DT contributed to the Appendices and to manuscript revision. ES, RB, FG, EC all contributed to manuscript critical revision. All authors read and approved the final manuscript.

## Authors' information

**SP**: MB BS BSc MRCP EDICM PhD, Consultant Cardiologist and Intensivist, Adult Intensive Care Unit, Royal Brompton Hospital, London, UK – Acute Cardiac Care Working Group – European Society of Cardiology (Nucleus Group Member) – British Society of Echocardiography Working Group on Intensive Care Ultrasound (Nucleus Member) – Intensive Care Society (UK) Education and Training Committee – WINFOCUS Echo-ICU Group (Member)

**GV**: MD, Intensivist, Anesthesiologist – Dept. of Anesthesia and Intensive Care – Fondazione IRCCS Policlinico San Matteo, Pavia, Italy – WINFOCUS Echo-ICU Group, (Secretary) – Faculty of the WINFOCUS UltraSound Critical Management Course (USCMC) – Director of the SMART Echocardiography for Intensivists Course

**ES**: Consultant, MD, PhD, DMSc – Dept. of Anaesthesiology and Intensive Care at Aarhus University Hospital, Skejby, Denmark – EACTA Echo Committee, member – WINFOCUS Echo-ICU Group (Member) – Member of WINFOCUS Directory Board

**RB**: MD, Scientific Employee at Hospital of the Johann Wolfgang Goethe-University, Frankfurt am Main, Germany – Consultant Anesthetist and Internist, EDIC – Main Scientific activity: Ultrasound research and development for Anesthesia and Critical Care Medicine – WINFOCUS Echo-ICU Group (Member) – Member of WINFOCUS Scientific Committee

**EC**: MD, Cardiologist, Anesthesiologist – Unit of Anesthesia and Intensive Care, "A. De Gasperis" Cardiologic Department – Niguarda Cà Granda Hospital, Milan, Italy – Faculty of the WINFOCUS UltraSound Critical Management Course (USCMC) – WINFOCUS Echo-ICU Group (Member).mailto:bilotta@tiscali.it

**FG**: MD, Director of Cardiothoracic Anaesthesia Dept. and ICU, Azienda Ospedaliera Universitaria Pisana – President of ITACTA, Italian Association of CardioThoracic Anaesthesiologists (Italian Chapter of EACTA,) – EACTA Echo Committee, member – WINFOCUS Echo-ICU Group (Member)

**DT**: MD MPH, Director of Critical Care Department of Anesthesia, Critical Care and Pain Medicine Beth Israel Deaconess Medical Center – Associate Professor of Anesthesia, Harvard Medical School Boston, MA, USA. – WINFOCUS Echo-ICU Group, (Chair) – Member of WINFOCUS Directory Board

## Appendix 1: Recommended theoretical syllabus[40,63,64,66]

This basic theoretical training is a prerequisite to any practical training in echocardiography.

### Physics and instrumentation

• The basic components of an ultrasound system

• Types of transducer and the production of ultrasound, with an emphasis on operator controlled variables

• Use of ultrasound controls

• An understanding of the frequencies used in medical ultrasound and the effect on image quality and penetration

• The interaction of ultrasound with tissue including biological effects

• The basic principles of real time and Doppler ultrasound including colour flow and power Doppler

• The recognition and explanation of common artefacts

• The safety of ultrasound and of ultrasound contrast agents

• Image recording systems [63]

### Ultrasound techniques

• Choice of echocardiographic modality (TEE vs. TTE)

• Choice of machine [67]

• Patient information and preparation

• Indications for examinations

• Relevance of ultrasound to other imaging modalities

• The influence of ultrasound results on the need for other imaging

• Scanning techniques including the use of spectral Doppler and colour Doppler

### Administration [63,64]

• Image recording

• Image storing and filing

• Reporting

• Medico-legal aspects – outlining the responsibility to practise within specific levels of competence and the requirements for training

• Consent

• The value and role of departmental protocols

• The resource implications of ultrasound use

## Appendix 2: Recommendations for performing a standard adult TTE on the ICU [63,68]

### Minimum dataset

The minimum dataset required for completion of a comprehensive TTE study is shown below. In all cases, recording of patient demographics is mandatory. Unless peri-resuscitation, and ECG recording is not readily available, this should always be applied. Where focused echocardiography is used, only part of the "minimum dataset" for a comprehensive exam is required, and this is described in detail in Appendix 5. Where TTE images are non-diagnostic, TOE may be indicated (see **Appendix 3**).

1. Identifying information

• Patient name

• A second unique identifier such as medical record number or date of birth

• Identification of the operator e.g. Initials

2. An ECG should be attached

3. 2D Views

• Parasternal long axis

• Parasternal short axis at the following levels

- aortic valve (base)

- mitral leaflet tips

- papillary muscles

• Apical four chamber

• Apical five chamber

• Apical two chamber

• Apical long axis

• Subcostal views to show the right ventricle, atrial septum and inferior vena cava

• Suprasternal view

4. M-mode or 2D measurements

• LV dimensions from the parasternal long axis or short axis view

- Septal thickness at end diastole

- Cavity size at end diastole

- Posterior wall thickness at end diastole

- Cavity size at end systole

- Aortic root dimension

- Left atrial dimension

5. Color Doppler mapping

• For the pulmonary valve in at least one imaging plane

• For all other valves in at least two imaging planes

6. Quantitative spectral Doppler

• Pulsed Doppler at the tip of the mitral leaflets in the apical 4-chamber view. Note E and A velocities, and E deceleration time

• Pulsed Doppler in the left ventricular outflow tract. Note systolic velocity integral

• Continuous wave Doppler across the aortic valve in the apical 5-chamber view. Note the peak velocity

• Continuous wave Doppler across the tricuspid valve. Note peak velocity.

• Pulsed or continuous wave Doppler in the pulmonary artery

7. Tissue Doppler imaging

• TDI (M-mode where views sub-optimal) of annular motion in the apical 4-chamber view. Basal free wall, septal, and RV.

Table 2 [see additional file 1] is an outline for a full, comprehensive TTE study, including the Minimum Dataset, with the inclusion of additional views and measurements. The table summarizes the minimum and additional data comprising the standard adult TTE study by view, modality, structure, measurements, and derived calculations. As described above, where focused echocardiography is required, appropriate sections of the "minimum dataset" should be performed, and the nature of the study clearly recorded in the patient record.

## Appendix 3. Recommendations for performing a standard adult TEE on the ICU [4,63]

### Minimum dataset

The minimum dataset required for completion of a comprehensive TEE study is shown below. In all cases, recording of patient demographics is mandatory. Unless peri-resuscitation, and ECG recording is not readily available, this should always be applied. Where focused echocardiography is used, only part of the "minimum dataset" for a comprehensive exam is required, and this is described in detail in **Appendix 5**.

1. Identifying information

• Patient name

• A second unique identifier such as CRN or date of birth

• Identification of the operator e.g. Initials

2. An ECG should be attached

3. 2D views

• Transgastric LV short axis

• Transgastric LV long axis

• Transgastric LVOT

• Transgastric RV long axis

• Deep transgastric

• Mid-esophageal four chamber

• Mid-esophageal bicommissural

• Mid-esophageal long axis

• Aortic valve short axis

• RV inflow/outflow

• SVC/IVC

• Pulmonary veins

• Pulmonary artery bifurcation

• Descending/arch/ascending aorta

4. M-mode or 2D measurements

• LV dimensions from the transgastric short axis view

- Cavity size at end diastole

- Cavity size at end systole

• Aortic root dimension

• Left atrial/mitral annulus dimension

• Right atrial dimension

5. Color Doppler mapping

• For the pulmonary valve in at least one imaging plane

• For all other valves in at least two imaging planes

6. Quantitative spectral Doppler

• Pulsed Doppler at the tip of the mitral leaflets in the mid-esophageal long axis view. Note E and A velocities, and E deceleration time

• Pulsed Doppler in the left ventricular outflow tract. Note systolic velocity integral

• Continuous wave Doppler across the aortic valve in trans-gastric LVOT view/deep transgastric view. Note the peak velocity

• Continuous wave Doppler across the tricuspid valve. Note peak velocity.

• Pulsed and continuous wave Doppler in the pulmonary artery

Table 3 [see additional file 2] describes the minimum dataset and additional data comprising the standard adult TEE study by, view, modality, structure, measurements, and derived calculations. As described above, where focused echocardiography is required, appropriate sections of the "minimum dataset" should be performed, and the nature of the study clearly recorded in the patient record.

## Appendix 4: Practical requirements for training in ICU echocardiography

Although training in ICU echocardiography is divided into levels of competence, as already described [Additional file 3] clear demarcation between the levels is not absolute, and the practicalities of obtaining training in echocardiography provide specific challenges to both the trainee and their supervisor. Where echocardiography fellowships of appropriate focus and duration are not available, WINFOCUS suggests a supervised modular approach to obtaining echocardiographic skills, comprising emergency, introductory, and intermediate stages. On completion of all modules, it would be anticipated that the practitioner would have performed the number of studies and achieved the competency to undertake a recognised accreditation examination [Additional file 3] through a national/international accrediting body. An outline of the modular structure is described [Additional file 4], followed by a description of training requirements at each level. The modules are described in detail in **Appendix 5**.

### 4.1 Introductory and emergency ICU echocardiography modules

The entry level modules for echocardiography will include focused TTE in the peri-arrest situation, aimed to make a diagnosis from a limited differential (FEEL [61]). An additional module will include an extended focused, short, systematic echocardiogram, incorporating basic dimensional measurements (FATE [30]). Training in such modules will be based on didactic teaching (course or internet-based) together with practical experience. It is likely that one or both of these peri-resuscitation modules will become a core competency in training ICU physicians of the future. Additional try level modules will cover the theoretical basis of echocardiography (see **Appendix 1**):

• Principles of US

• Principles of Doppler

• "Knobology"

• Pitfalls and artefacts

• Patient safety and consent

• Sono-anatomy of the heart

• Focused Echocardiographic Evaluation in Life support (FEEL)

• Focused Assessment with Transthoracic Echocardiography (FATE)

### 4.2 Basic ICU echocardiography modules

• Qualitative evaluation of the left ventricle

• Qualitative evaluation of the right ventricle

• Measuring IVC &/or SVC diameter

• Measurement of LVOT peak velocity and VTI

• Measurement of Cardiac Output

• Estimation of peak PA pressure

### 4.3 Intermediate ICU echocardiography modules

#### 4.3.1 Assessment of ventricular function

• Quantification of LV systolic function

• Quantification of RV systolic function

• LV diastolic function

• RV diastolic function

• Estimation of left atrial pressure

• Detection of myocardial ischaemia

#### 4.3.2 Assessment of valvular function

• The Aortic valve-AS/AR

• The Mitral valve-MS/MR

• The Tricuspid valve

• The Pulmonary valve

### 4.4 Echocardiography in clinical scenarios

There are conditions that occur in the ICU that require addressing specifically in a document concerned with ICU echocardiographic training which may be the subject of focused or comprehensive examinations. These include:

• Assessment of shock

• Hypovolemia, volume responsiveness and filling status

• Tamponade and pericardial disease

• The sepsis syndromes

• Effects of pre-load and afterload

• Hypoxemia

• Anatomical shunt

• Acute Cor Pulmonale (Pulmonary Embolism, ARDS)

• Complications of acute MI

• Echo in chest trauma

• Weaning failure from mechanical ventilation

• Hemodynamic measurements

Where a study performed is focused, rather than comprehensive, this should be stated in the report. The specific details regarding the basic echocardiographic modules for focused assessment in each of these clinical scenarios are addressed in **Appendix 5**.

#### Level 1: training and practice

• Practical training should involve carrying out regular echocardiography examinations in the critical care unit or echo department, with approximately 5 examinations performed by the trainee (under supervision) per week

• A minimum number of examinations should be undertaken – 50–100 is deemed as appropriate number of examinations required for a trainee to become competent. However different trainees will acquire the necessary skills at different rates and the end point of the training programme should be judged by an assessment of competencies.

• Examinations should encompass the full range of pathological conditions and practical procedures listed in **Appendix 5**

• A logbook listing the types of examinations undertaken should be kept

• Training should be supervised either by someone who has obtained at least Level 2 competence in critical care echocardiography themselves

• Trainees should attend an appropriate theoretical course and should be familiar with the published literature

• During the course of training the competency assessment should be completed as this will determine in which area or areas the trainee can practise independently, i.e focused echocardiography in resuscitation

#### Level 2: training and practice

• Practical training should include at least 1 year of experience at Level 1 with a minimum of one session or equivalent per week

• A logbook listing all examinations undertaken should be kept

• A level 2 practitioner should have the competencies to undertake a recognised accreditation

• The trainee should be part of a departmental continuous quality improvement program

• Supervision of training should be undertaken by someone who has achieved at least Level 2 competence in critical care echocardiography, has had at least 2 years' experience at that level and would normally be of consultant status

• A Level 2 practitioner will be able to accept referral from Level 1 practitioners

• A level 2 trainee should be involved in echocardiographic research with the aim of advancing the knowledge base of the field

## Appendix 5: clinical practice of echocardiography in the ICU

### 5.1 Ventricular function

The critical care echocardiographer must take great care in the interpretation of ventricular function. It must be taken in the context of the clinical status of the patient, and the level and degree of inotropic support. Hence, the normal range values that apply in the outpatient setting may not be relevant to the ICU[69].

#### 5.1.1 Systolic function & regional wall motion abnormalities

Myocardial damage causing left and/or right ventricular systolic dysfunction can result from a number of pathologies seen on the ICU including:

• Acute coronary syndromes

• Septic myocardial depression

• Acute myocarditis of other etiologies

Other conditions can cause systolic ventricular dysfunction, without necessarily causing myocardial damage i.e. excessively increased afterload to the right ventricle resulting in decreased systolic performance. When assessing ventricular function, each ventricle should be considered alone, and also in conjunction with the other ventricle.

##### Left ventricular systolic function. Principles of examination

LV systolic function can be evaluated using echocardiography (TTE and TEE) in many ways; however, each has its limitations:

• Shortening fraction by M-mode examination – only assesses basal systolic function and is load dependent

• Fractional area change (FAC) – subject to geometric assumptions and is load dependent

• Ejection fraction (EF) – subject to geometric assumptions and is load dependent

• Tei index – is also load and ventricular activation dependent [70,71]

• Long axis function and tissue Doppler Imaging (TDI) – may assist (when used in conjunction with trans-mitral Doppler) in the assessment of LV systolic function [72,73]

##### Right ventricular systolic function. Principles of examination

RV systolic function can also be evaluated using echocardiography, and should not be overlooked in the critically ill:

Tricuspid annular plane excursion during systole (TAPSE, or RV long axis function) obtained by M – mode or TDI of the tricuspid annulus is well validated in assessment of RV systolic function. Problems with correct alignment using TEE may be overcome using anatomical M-mode [74]

##### Regional wall motion abnormalities

Regional myocardial function plays an important role in diagnosis of myocardial ischemia – a new regional wall motion abnormality suggesting the onset of new ischemia. Regional wall motion abnormalities are detected using various echocardiographic tools, including:

• 2D assessment with wall motion scoring indices

• Long axis annular motion and the demonstration of post-ejection shortening

• Tissue Doppler and myocardial strain (for the experienced echocardiographer)

Standard assessment is with 2D detection of regional wall motion abnormalities, however, these occur relatively late in the ischemic cascade. Of relevance to the ICU setting, where TTE is used to detect regional wall motion abnormalities, echocardiographic contrast agents improve endocardial border definition and hence diagnostic sensitivity[75], however, their use may be precluded in the critically ill.

#### 5.1.2 Diastolic function

There is much debate in the literature regarding the definition of diastolic dysfunction; however, for the purposes of ICU echocardiography, this should be taken to mean abnormalities of ventricular filling that may be independent of systolic dysfunction[76,77]. As a primary reason for ICU admission, diastolic heart disease is extremely rare, however, the finding of abnormalities of diastolic filling patterns in the critically ill is not unusual, and the diagnosis has relevance to the haemodynamic management of such patients.

##### Left ventricular diastolic function. Principles of examination

This can be assessed using TTE and TEE. Necessary for interpretation are:

• Trans-mitral Doppler flows (PW)

• Pulmonary vein Doppler flows (PW)

• Tissue Doppler Imaging (TDI)

• (M-mode colour Doppler of mitral inflow)

Abnormalities in LV filling may occur in many conditions seen in the ICU, including aortic stenosis, hypertension, tamponade, pulmonary embolism, and severe LV systolic dysfunction. Any interpretation of LV filling patterns must therefore be made in the context of the full echocardiographic study.

##### Right ventricular diastolic function. Principles of examination

Although less well studied, there are features of right ventricular function that suggest the diagnosis of RV diastolic disease. These include:

• Dominant diastolic flow in the SVC or IVC (equivalent to a dominant y descent)

• Presence of a significant A wave on pulmonary arterial Doppler throughout the respiratory cycle [78]

• Inappropriate (for age) dominant E wave on trans-tricuspid filling

##### Relevance to the ICU setting

The finding of diastolic filling abnormalities (right and/or left-sided) in the critically ill patient should signal caution in the interpretation of filling pressures measured by standard invasive means, as such patients may require relatively high filling pressures to maintain cardiac output. Where very short trans-mitral filling patterns are seen:

• The filling pattern may be summation at high heart rates

• If E wave filling only, a high heart rate may be needed to maintain cardiac output

• If A wave filling only (particularly where dominant E wave filling is expected) a reason for suppression of early diastolic filling should be sought

• If pacemaker programming is changed to optimize filling in restrictive disease, the echo should be repeated to ensure ventricular filling has not been further compromised

### 5.2 Hypovolemia, volume responsiveness and filling status

Inadequate circulating volume is a common feature of many of the syndromes encountered in ICU[79] and optimization of volume status is crucial and often challenging [80,81]. The decision regarding "volume status" and fluid challenges is perhaps the most frequent decision required in ICU and the correct answer depends on accurate prediction of preload-responsiveness (or volume-responsiveness, VR) [82]. Here, echocardiography may be useful.

#### Principles of examination

Echocardiography enables assessment of the patient's volume status which is complementary to, and may be more reliable than measurements performed by traditional invasive hemodynamic monitoring [83-85]. In the ICU, when images are sub-optimal, TEE may allow for a more accurate assessment of intracardiac flows, heart-lung interactions, and superior vena cava distensibility than TTE, however, the information available from TTE often is adequate. Echocardiographic volume status assessment is based on static findings (single-measure dimensions and flows) and dynamic indices targeted to VR determination (variation in flows and dimensions after dynamic maneuvers: spontaneous or mechanical respiratory loading, passive leg raising, and/or fluid challenge).

In the ICU setting, the following considerations must be taken into account:

• Assessment of VR requires measurement of multiple parameters

• LV or RV end-diastolic dimensions are unreliable predictors of VR

• The effects of Intermittent Positive Pressure Ventilation (vs. spontaneous ventilation) must be considered when looking at changes due to respiration

• Preload responsiveness Indices have been validated mainly in septic shock and perioperative patients

• Where a patient is not in sinus rhythm or is ventilated with intermittent spontaneous respiratory activity, assessment of VR by means of heart-lung interactions study may not be accurate. Here, assessment of the effect of passive leg raising may be the right tool [86-89]

• Non echocardiographic VR heart-lung interaction-based indices (such as Pulse Pressure variations) may present false positives (especially in severe RV failure) which can be easily detected by echocardiography.

#### Detection of severe hypovolemia

The following parameters have been suggested to indicate severe hypovolemia in the critically ill, and should be measured routinely when assessing for volume responsiveness using echocardiography:

• Small hyperkinetic LV (in the presence of a normal RV), with cavity end-systolic obliteration [90] (but caution in patients with severe valvular regurgitation, excessive inotropic support or left ventricular hypertrophy)

• LVEDA < 5.5 cm^2^/m^2 ^BSA [91]

• Small IVC with inspiratory collapse, in spontaneously breathing patients [92], or small IVC at end expiration [93]with variable (depending on adaptation to ventilator) respiratory change, in mechanically ventilated patients [94,95]

#### Screening for low tolerance to volume loading

• Severe Right Ventricular Dysfunction (RVEDA/LVEDA > 1) [91]

• Signs of systemic venous congestion (dilated fixed-diameter IVC and SVC) in the absence of tamponade

• High estimated LV filling pressures – but note caution with restrictive ventricular disease (section 5.1.2)

#### Heart-lung interactions

In fully mechanically ventilated patients in sinus rhythm, predictors of VR are:

• Superior Vena Cava Collapsibility Index > 36%[96]

• Inferior Vena Cava Distensibility Index > 18%[97] or 12%[98]

• LV Ejection variations: LVOT Vmax Variations > 12%[99], LVOT VTI Variations > 18% [100]

#### Passive leg raising

In spontaneous/assisted ventilation, or in case of rhythm other than sinus, passive leg raising can be used as an endogenous fluid challenge to assess VR [86]: an increase in LVOT peak flow > 12% has been shown to predict VR [88,89].

#### Assessment of fluid loading maneuvers

This can be done by means of:

• Cardiac Output measurement (at LVOT or at AV orifice) [101,102] – an increase will demonstrate the effectiveness of the fluid challenge

• LVEDA – increase will parallel the reached increase in LV preload and with maintained systolic function, lead to an increase in stroke volume [87,103,104]

• Doppler estimations of LV filling pressures (section 5.2.2) – to determine tolerance to the fluid challenge

Note: raised pericardial pressure and/or pleural pressure may lead to physiological "underfilling" of the ventricle(s) demonstrated using echocardiography, even when pressures measured by standard invasive means are increased. In assessment of filling, it is the trans-myocardial pressures that matter, not the absolute values measured.

### 5.3 Tamponade and pericardial disease

The pericardium comprises two layers (visceral and parietal), with 5–10 ml pericardial fluid between the layers, and serves to restrain the four cardiac chambers within a fixed volume. There are a number of conditions where inflammation and/or accumulation of fluid in the pericardial space may cause abnormal hemodynamics on the ICU include:

• trauma

• cardiac surgery

• congestive cardiac failure

• myocardial infarction

• inflammatory/infective pericarditis

Most inflammatory conditions affect both layers, and may be associated with the accumulation of fluid in the pericardial space, in addition to the development of pericardial constriction.

#### Principles for examination of the pericardium & pericardial space

The pericardium and pericardial space should be assessed in all views using TTE where possible. Following cardiac surgery, or where images are sub-optimal, a TEE may be indicated (see below). Of note:

• The thickness of the pericardium and depth of any pericardial fluid should be measured (TEE thickness > 3 mm 95% sensitive & 86% specific) [105]

• The presence of any loculations should be noted. In particular, where percutaneous drainage is being considered, subxyphoid views should be obtained and suitability for drainage assessed

• Left ventricular function should be assessed – particularly with respect to motion of the posterior and septal walls (PLAX)

• Using M-mode on the PLAX or RVOT view, RV diastolic collapse may be demonstrated where there is tamponade

• Doppler examination of the following should be performed in all cases, in particular noting variation with respiration:

- trans-mitral and trans-tricuspid

- trans-pulmonary

- LVOT

• Finally, the diameter of the SVC should be measured, together with variation with respiration, and the venous return to the right heart recorded using PW Doppler

#### Tamponade

The commonest pericardial disease seen in the ICU is tamponade. Here, the degree of hemodynamics compromise caused by accumulation of pericardial fluid is related to the intra-pericardial pressure, which in turn will depend upon both the volume and rate of accumulation [106]

Features of tamponade may not be typical in the ICU [107]:

• Phasic changes in flow with inspiration and expiration are reversed in positive pressure ventilation)

• The presence of classical echocardiographic changes depend upon a global collection with equal transmission of increased intrapericardial pressures. Under certain circumstances (i.e. post-cardiac surgery with localized collections, or right ventricular hypertrophy with a non-compliant, hypertrophied RV [108]), these changes may not be seen.

• Following cardiac surgery, not only are these features often absent [109] but collections are small and easily missed using TTE. Here, if time permits, a TEE should be performed to determine the presence of a collection, and this, together with clinical features of hemodynamics compromise (or oliguria) is sufficient to make the diagnosis.

• Tamponade can also occur in the absence of a pericardial collection, such as with large pleural effusions [110].

#### Thus, it should be remembered that tamponade is primarily a clinical, not an echocardiographic diagnosis

#### Pericardial constriction

This is an unusual indication for ICU echo. Where a patient has elevated right heart filling pressures, resistant to diuresis and a history suggestive (previous TB, CABG or mediastinal radiotherapy) the diagnosis may be suspected. The physiological changes result from fusion of the pericardial layers forming a sac of restricted capacity. Thus, there is interdependence of filling of the LV and RV, which occurs in early diastole. Clinical features include:

• Elevated JVP which rises on inspiration (reversed with IPPV)

• Peripheral edema/ascites

• Pulsus paradoxus (may not be present)

Characteristic echo findings include thickened pericardium, preservation of ventricular long-axis function (TDI), marked respiratory variation in trans-valvular Doppler velocities, and dominant × descent on SVC/IVC venous filling [111]. Where a patient is operated for pericardial constriction, post-operative echocardiography often reveals underlying restrictive ventricular disease.

### 5.4 Sepsis syndromes

Echocardiography can play a key role in the management of the septic ICU patient both by excluding cardiac causes for sepsis, and by guiding haemodynamic management of those patients in whom sepsis reaches such a severity to jeopardize cardiovascular function and survival.

#### 5.4.1 Assessment in septic shock

##### General principles

Sepsis and septic shock (SS) are the most common causes of ICU mortality [112,113]. SS is one of the most complex hemodynamic failure syndromes, and may imply derangement of all of the three mainstays of cardiovascular homeostasis, each one to a variable degree: absolute or relative reduction in central blood volume, severe peripheral vasodilatation, and RV/LV myocardial failure [114,115]. Even if echocardiography is not available as a continuous monitoring tool, repeated bedside assessment at each hemodynamic deterioration or significant therapeutic intervention is advisable [12], allowing for prompt recognition and correction of the causes of cardiovascular instability [116,117]. Echocardiographic findings should be integrated with clinical data and other monitoring information, especially with those concerning the adequacy of peripheral tissue perfusion. TEE enables for a complete assessment, also detailing heart-lung interactions and fine volume responsiveness evaluation. TTE, where adequate transthoracic views can be achieved usually provides adequate information, allowing for less invasive and more readily repeatable assessment, especially once key hemodynamic features have already been demonstrated.

##### Echocardiographic approach and key findings

Each echocardiographic assessment should seek for the following situations, thus guiding fluid therapy and inotropic/vasoconstrictor support institution and titration:

###### 1) Low output state

Due to peripheral flow distributive derangements, normal values of cardiac index (CI) should not be considered necessarily adequate in SS. However, determination of CI can provide the following information:

• An indicative value to class the patient into ranges of CI (low, normal, high)

• A reference for subsequent determinations after therapeutic intervention or clinical changes

###### 2)Inadequate central blood volume

A state that can be easily and thoroughly assessed (section 5.2). Echocardiographic assessment of a patient in the initial phase of SS will generally reveal hypovolemia with hyperkinetic biventricular systolic function, unless underlying chronic cardiac dysfunction, other sepsis-triggered cardiac derangements co-exist, or aggressive mechanical ventilation (hindering RV function in the context of ARDS/pneumonia) is used. Where the shock state is volume-resuscitated, echocardiography can be used to determine VR[118] (section 5.2).

###### 3) LV systolic dysfunction

Sepsis-related LV systolic dysfunction is a well-recognized[119] phenomenon. Both global and regional systolic wall motion abnormalities can be found [120,121], and a hypokinetic state (low cardiac index associated with reduced EF) described in up to 60% of SS patients[122]. There is no LV "adaptation" to this systolic function reduction by an increase in chamber dimension. Therefore, no significant LV dilatation is to be expected in a previously healthy septic-depressed LV[123,124]. Sequential determinations of LV function (section 5.1.1) may allow assessment of LV recovery in survivors[12,125].

###### 4) RV systolic dysfunction

RV systolic dysfunction may develop in up to one third of patients with SS, either in isolation, or associated with LV dysfunction[124-126] Intrinsic depression of RV myocardial function is detected as RV hypokinesia (see section 5.1.1), and semi-quantitatively appreciated as variable degree of RV dilatation (with RVEDA/LVEDA ratio measurement). When RV afterload is increased (due to ARDS, mechanical ventilation or pharmacological agents) on the background of an already impaired RV or not, acute cor pulmonale can be diagnosed using echocardiography [127,128].

###### 5) Low peripheral vascular tone

Echocardiography offers the tools to determine arterial vascular resistance [129], but is cumbersome. In clinical practice, sepsis-related vasodilatation is diagnosed using exclusion criteria based on clinical and echocardiographic findings:

• Persistence of hypotension despite adequate preload and preserved (or pharmacologically normalized) biventricular systolic function.

##### Of particular note in the ICU

• Always screen for pre-existing cardiac dysfunction

• Where ventricular dysfunction is found in a patient with SS, an ECG may help to distinguish between acute coronary syndrome-determined dysfunction (triggered by sepsis) from true sepsis-related myocardial dysfunction. Cardiac troponins may not [130].

• LV dysfunction can be masked by sepsis-associated vasodilatation and preload inadequacy: always reassess LV function after preload and afterload optimization.

• Assessment of LV (and RV) systolic function should be performed after correction of hypovolemia.

• Extreme tachycardia (or tachyarrhythmia) can make volume status and bi-ventricular function difficult to assess. Here, assessment of the vena cavae may be helpful. Re-assessment after sinus rhythm/acceptable heart rate restoration will give more reliable estimate of myocardial dysfunction, if present, and outline a different volume status situation.

#### 5.4.2 Cardiac source of sepsis

Infective endocarditis (IE) is a microbial infection of intracardiac structures facing the blood. It can be encountered in ICU patients both as cause of admission or as acquired infection leading to a septic state with no evident focus.

##### Infective endocarditis on native or prosthetic valves

The diagnosis of IE is defined on the basis of a well established set of diagnostic criteria of which echocardiography is one of the major [131,132]. IE is a severe disease with a high mortality, ranging from 20 to 25% and up to 45% in patients then admitted to ICU[133,134]. Echocardiography contributes to IE diagnosis, allowing for assessment of severity, and has a pivotal role in management and decision making [135].

##### Principles of examination

• Three echocardiographic findings are important in establishing diagnosis of IE:

- Mobile echo dense mass(es) attached to valvular or mural endocardium, or to implanted material

- fistulae or abscess formation

- new disruption or dehiscence of a prosthetic valve (paravalvular leak)

• TEE has greater sensitivity for detection small vegetations and on mitral valve IE than TTE. Both techniques reach high specificity in equal manner[136].

• The clinical context influences the diagnostic capability of TTE and TEE: while with low IE pre-test probability a negative good-quality TTE can exclude the diagnosis, TEE should be performed on all TTE negative cases with a high index of clinical suspicion. In mechanical ventilated ICU patients TEE is almost invariably needed [137].

• TEE is mandatory in the assessment of suspected prosthetic valves IE, and in TTE positive cases to identify major valvular complications and guide surgical planning [138].

##### IE on indwelling central venous catheters or implantable devices

This is uncommon – probably due to the lack of the hemodynamic factors usually associate with the pathogenesis of IE (flow turbulence, high pressure gradients) [139], however, the incidence is increasing with increasing patient intervention. In the ICU, the diagnosis should be considered in septic patients with no other clear infective focus, particularly if evidence of septic pulmonary embolism exists [140]. Besides searching for vegetations on any catheter, from superior vena cava to its implantation on the myocardium, the examination (TTE and TEE) should focus on the search of infection on the right-sided heart valves [139,141-143]. Septic thrombus on temporary central venous catheters in ICU patients[144] and right heart endocarditis following pulmonary heart catheterization have also been described[145]. Finding of masses on central venous catheters should prompt to consider non-septic thrombosis as differential diagnosis.

##### Of note

• Clinical presentation of IE in the acutely ill can be highly variable

• Echocardiography alone cannot be used to make diagnosis of endocarditis: a combination of clinical-instrumental-microbiological criteria is required

• A third to a half of IE develops in absence of pre-existing cardiac pathology or prosthetic devices

• Keep high suspicion for endocarditis in ICU bacteremic patients with unknown septic focus, especially if patients with prosthetic valves, implantable devices, or known significant native valve pathology

• In severely damaged native valves (especially rheumatic), clear identification of small vegetations may be very difficult. Differential diagnosis between prosthetic valve IE and non-obstructive thrombus, or between bioprosthetic valve IE and degeneration, can be very challenging

• Viewing of vegetations on implantable devices (mass or sleeve-like) may be difficult, due to artefacts coming from the device itself. Small fibrin strands may represent a difficult differential diagnosis

### 5.5 Effects of preload and afterload

All indices of LV/RV systolic and diastolic function currently used in clinical practice are heavily load dependent: findings must always be interpreted in the context of drugs/situations affecting afterload[146] (vasodilators, anesthetics, sedatives, vasoplegia, effects of airway pressure on pulmonary circulation) (see sections 5.1, 5.12.1 and 5.12.3) and in the context of actual preload and filling status (see sections 5.2 and 5.12.2), also by integrating echocardiographic data with invasive monitoring, when available.

### 5.6 Hypoxemia

Echocardiography may be used to assist in the diagnosis and management of hypoxemic patients on the ICU in many ways, including:

• Differential diagnosis between cardiogenic and respiratory pulmonary edema[147]

• Assessment of secondary effects of pulmonary pathology on cardiac performance

• Diagnosis of anatomical shunt (intra-cardiac or intra-pulmonary)

• Assessment of a low cardiac-output state contributing to arterial hypoxemia

In addition, transthoracic lung ultrasound may be helpful, but is beyond the scope of his document.

#### 5.6.1 Cardiogenic pulmonary edema

Echocardiographic demonstration (TTE or TEE) of a cardiac cause for pulmonary edema ultimately relies on detection of pulmonary venous hypertension and its underlying cause [55,148], by:

- LAP estimation (see section 5.12.2)

- Assessment of LV function

- Assessment of left-sided valvular pathology

Where the LAP is high and venae cavae suggest adequate filling, in the absence of any relevant cardiac pathology, simple volume overload may be diagnosed.

##### Of note

• TTE assessment is usually adequate to make the diagnosis

• If a TEE is indicated, the hypoxemic patient will probably require intubation

• The cause of pulmonary edema may be dynamic and/or occur on ventilatory weaning (i.e. due to ischemia, MR, hypertension). Here, a focused/targeted echocardiographic study (or stress echo) should be considered at the time of deterioration (see section 5.11)

• Isolated diastolic dysfunctionis extremely rare as a cause of acute pulmonary edema in ICU [149,150]

• Multiple causes of hypoxemia may coexist

• Unilateral pulmonary edema mimicking pneumonia has been described as consequence of eccentric mitral regurgitation [151,152]

#### 5.6.2 Pulmonary Embolism (see section 5.7)

#### 5.6.3 Acute Respiratory Distress Syndrome (ARDS)/Acute lung injury (ALI) (see section 5.7)

#### 5.6.4 Anatomical shunt

Hypoxemia unexplained by pulmonary clinical-radiological findings may be due to intra-cardiac or intra-pulmonary shunting (or both). In ICU many factors increasing right sided intracavitary pressures can trigger or exacerbate the effects of such condition in case of *intracardiac shunt *(CPAP, intermittent positive pressure ventilation, acute tricuspid regurgitation, acute pulmonary hypertension, RV AMI, tamponade) [153-158]. *Intrapulmonary shunt*, unaffected by such hemodynamic factors, has been described in thoracic trauma, liver cirrosis [159]. The choice of technique depends on clinical context, quality of subcostal TTE imaging, and the need for detection of intrapulmonary shunt. TEE has superior diagnostic accuracy [160,161]. The diagnosis lies on:

- a positive agitated saline contrast study: microbubbles passage into left atrium, either immediate (patent foramen ovale, PFO or atrial septal defect, ASD) or delayed (intrapulmonary shunt, microbubbles through pulmonary veins) [162]

- direct visualization of PFO, ASD

- color-Doppler demonstration of right to left flow through PFO, ASD

##### Of note

• Provocative maneuvers (ie Valsalva) may be required to exclude PFO

• A high LAP may mask an intracardiac shunt [163]

• A significant right to left shunt may lead to overestimation of non-echocardiographic CO measurements

### 5.7 Acute cor pulmonale

Acute cor pulmonale is a life threatening condition characterized by a sudden severe increase of right ventricular afterload, determining a hindrance to RV ejection. Major causes of acute cor pulmonale are represented in ICU by acute pulmonary embolism and mechanical ventilation during ARDS.

#### 5.7.1 Pulmonary embolism

Massive pulmonary embolism (PE) is a cause of Acute Cor Pulmonale (ACP) encountered in ICU. Only direct visualization of thrombi in the pulmonary arteries enables a definitive diagnosis [164,165], however, in the majority of cases, echocardiography provides only indirect signs of PE [166,168], represented by:

- pulmonary hypertension at CW Doppler on tricuspid regurgitation

- signs of RV systolic overload (septal dyskinesia)

- signs of diastolic overload: (RV enlargement: RVEDA/LVEDA > 0.6)

- RV free wall hypokinesia

- Moderate to severe tricuspid regurgitation

Bedside echocardiography can nevertheless greatly aid clinical decision making in patients with acute PE [168] and monitor response to therapy [169]

##### Of note

• Differential diagnosis with RV AMI can be made on clinical context, absence of pulmonary hypertension, and coexistence of LV postero-inferior hypokinesia

• Gradation of RV dilatation by ventricular diastolic area ratio may be misleading in patients with coexisting dilated cardiomyopathy.

• Pre-existing chronic pulmonary hypertension may be suggested by RV hypertrophy, RA dilatation, and systolic pulmonary arterial pressure greater than 60 mmHg [170,171]

#### 5.7.1 ARDS/ALI

Where radiographic appearances suggest acute pulmonary edema, demonstration of normal left-sided filling pressures may suggest the diagnosis of ARDS/ALI [172]. Of note, however, the two conditions may co-exist. Where the cardiogenic component is dynamic (see above) the diagnosis is particularly challenging, and targeted echocardiographic examinations performed in conjunction with standard hemodynamic monitoring may be indicated.

Where the diagnosis of ARDS/ALI is made, mechanical ventilation may have unfavorable effects on RV function, due to lower lung compliance and secondary increase in alveolar pressure and pulmonary vascular resistances [173-175]. Ouvert acute RV failure can then develop and can be reliably assessed using echocardiography [176,177]. Due to introduction of more lung-protective ventilatory strategies this phenomenon is less frequently seen [178], however, a mild degree of RV dysfunction is not uncommon [178]. Recent evidence also suggests a role of echocardiography in the choice of ventilatory strategy in ARDS [179,180]

### 5.8 Complications of acute myocardial infarction

Coronary artery disease is the commonest acquired heart disease in adults, resulting in a reduction or interruption in coronary flow, with effects on ventricular and valvular function. The major complications relevant to the ICU setting that result from acute MI include acute MR, infarct expansion and ventricular septal rupture, right ventricular infarction, ventricular thrombus, and free wall rupture. Of note, almost all complications of acute myocardial infarction (AMI) may be diagnosed using 2D and color flow echocardiography [181]. The current ACC/AHA Guidelines for Clinical Application of Echocardiography in the diagnosis of acute myocardial ischemic syndromes are shown below[182]:

Class I

• Diagnosis of suspected acute ischaemia or infarction not evident by standard means

• Measurement of baseline LV function

• evaluation of patients with inferior MI and clinical evidence suggesting possible RV infarction

• Assessment of mechanical complications and mural thrombus (TEE indicated when TTE studies are not diagnostic)

Class IIa

Identification of location/severity of disease in patients with ongoing ischaemia

Class III

Diagnosis of AMI already evident by standard means

#### 5.8.1 Regional wall motion abnormalities

There is a recognized cascade of echocardiographically detectable abnormalities that occur as a result of myocardial ischaemia which correlate with the degree of flow reduction in the coronary arteries. Many of these occur before the onset of ECG abnormalities and are readily detectable in the ICU setting. This sequence of events is [183]:

• Normal ventricular function

• Perfusion abnormalities (detected with perfusion contrast agents)

• Abnormal long axis function (delayed onset and normal duration of contraction persisting into diastole (M-mode or TDI) associated with suppressed early transmitral filling on Doppler)

• regional wall motion abnormalities (2D echo)

• ECG changes

There is extensive literature regarding regional wall motion abnormality scoring and the relevant coronary artery involvement (section 5.1, above). Where possible the segmental approach to scoring regional wall motion changes following acute myocardial infarction should be used. Of note, these regional wall motion changes detected using 2D echo occur relatively late in the ischemic cascade. The earlier echo findings of ischaemia (particularly related to long axis function) are particularly useful in the ICU setting, however, there are particular considerations for this patient group, including:

• Echo contrast may be contraindicated (pulmonary hypertension, severe LV disease)

• Images may be suboptimal for accurate interpretation of TDI – here, M-mode of the mitral/tricuspid annulus is usually achievable

• Where there is significant LVH and excessive inotropic support, these echo findings may prove useful as an early indicator of induced ischaemia

• The development of these changes post-operatively or on attempting weaning from respiratory support may indicate significant coronary artery disease

• TEE may be useful in visualising the first few centimetres of the coronary arteries (particularly post-aortic root surgery) and identifying high velocities associated with restriction of coronary flow.

#### 5.8.2 Acute mitral regurgitation

Suspect acute mitral regurgitation (MR) post-AMI with hemodynamics compromise, pulmonary edema and pan-systolic murmur. The etiology of acute MR is readily detected using TTE +/- TEE and may be due to papillary muscle rupture, or LV dysfunction resulting in MV dysfunction[181]. Where appropriate urgent surgical referral is indicated.

• Partial/complete papillary muscle rupture

- Usually postero-medial papillary due to single coronary artery blood supply (may visualize disrupted head on 2D echo – but unusual)

- Color flow mapping helpful in detecting jet presence and direction (usually eccentric, anteriorly directed with posterior papillary muscle rupture)

- TEE may be necessary to determine anatomy accurately

• Infarction of the LV wall with disruption of normal MV coaptation

- Often posterior leaflet restriction (posteriorly directed jet)

- Jet may be central/eccentric

- Regurgitation may be very dynamic, depending upon loading conditions and ongoing ischemia

Of note, in the ICU setting ischemic MR may be difficult to diagnose [11]:

• TTE images may be suboptimal, especially if patient ventilated and in pulmonary edema. Here, TEE may be indicated to make the diagnosis

• Grading the severity of MR: account must be taken of the pre-load and after-load, and if necessary loading and inotropic conditions altered.

• In severe MR the characteristic regurgitant jet recorded on continuous-wave (CW) Doppler may be short and of low velocity (< 3 m/s), and in the most severe cases may not be visualized using colour flow. Here, PW Doppler (showing laminar flow) or ventricular angiography may be necessary.

• Ischemic MR is a recognized cause of failure to wean from ventilatory support in the ICU. Where suspected, a targeted echocardiographic study performed when the patient is clinically compromised is indicated. If not possible, stress echocardiography (using Dobutamine, +/- volume loading and pressor agents) may be necessary.

#### 5.8.3 Ventricular septal rupture

This is a relatively uncommon complication following the introduction of effective and prompt thrombolysis and/or percutaneous coronary intervention [181]. As with acute, severe MR, ventricular septal rupture (VSD) should be suspected post-AMI where patients present with hemodynamic compromise, pulmonary edema and a pan-systolic murmur.

• Does not follow congenital patterns of VSD – may occur at any point along the interventricular septum – non-standard views may be needed

• The path of the VSD may be eccentric (or multiple) – first identify the defect using color flow Doppler, and subsequently perform 2D imaging

• Apical VSDs may be particularly difficult to identify using TEE

• Additional assessment should include: biventricular function and pulmonary arterial pressures

#### 5.8.4 Right ventricular infarction

The index of suspicion of RV infarction should be particularly high in patients with an inferior AMI due to occlusion of the proximal RCA. Echocardiographic features include [184]:

• Impairment of RV function (as in LV ischaemia) assessed using M-mode and TDI of the RV LAX – may be relatively subtle

• Dilatation and hypokinesis/akinesis of the RV are less common (assess using 2D and M-mode and TDI of the RV LAX) – indicate more extensive infarction

• Associated secondary tricuspid regurgitation

• Falling PA pressures (from TR) may indicate progressive RV dysfunction

• High right-sided pressures may be indicated by right-left shunting through a PFO

• LV systolic function usually well-preserved (may see inferior WMA)

#### 5.8.5 Pericardial fluid

The pericardial space should be imaged as described in section 5.3. A small amount of fluid in the pericardial space is not uncommon following a trans-mural AMI, and rarely causes hemodynamic compromise. Rupture of the free wall of the left ventricle may occur (rarely since introduction of thrombolysis) and will result in a very rapidly accumulating collection in the pericardial space – and is almost invariably fatal.

### 5.9 Echocardiography following chest trauma

Echocardiography either TTE or TEE may be performed in the emergency room department to assess the effects of blunt and penetrating chest trauma. The range of findings and echocardiographic indications are beyond the scope of this paper, which focuses specifically on ICU echocardiography. Indications for emergency assessment are widely discussed[30,61,185] and the use of echocardiography in this setting is undisputed.

There are several considerations in the echocardiography of patients with chest trauma that are admitted to the ICU. First, TTE has a relatively low diagnostic yield compared with TEE[186]. Second, where tamponade is suspected, TTE may provide the diagnosis rapidly and is particularly useful in the diagnosis of aortic disruption[187,188]. Delayed tamponade is not uncommon (for the echo features, see section 5.3). Third, a huge range of abnormalities have been described following chest trauma [189,190], and therefore where time permits, a comprehensive study should be performed. These findings include:

• Hemopericardium – the hemodynamic consequences depend upon the site, rate and volume of collection [191,192]. Small, rapidly accumulating collections may be hemodynamically very significant. Beware late tamponade.

• Pleural collections – echocardiography may demonstrate the presence of a pleural collection. Where associated with a pericardial collection, high intra-pleural pressures may be transmitted to the pericardium resulting in tamponade which may be resolved by evacuation of the pleural and pericardial fluid

• Aortic valve disruption – any type of disruption of the valve can occur both from penetrating and non-penetrating injury. Acute disruption may result in catastrophic regurgitation[193]. Late effects may be progressively worsening regurgitation, valve degeneration and stenosis

• Aortic disruption – may occur due to blunt or penetrating chest trauma, or deceleration injuries. TEE has a high sensitivity and specificity for diagnosis of aortic disruption, but note may miss disruption of arch or branches [194]

• VSD – often atypical position. Determine with color flow first, then anatomy with 2D

• Coronary artery disruption – may see associated evidence of ischemia (section 5.1) and/or fistulae

• Mitral valve disruption – penetrating wounds to the chest (knife or bullet) have been associated with MV regurgitation. The anatomical abnormality may be missed using TTE

• Myocardial contusion – may see regional wall motion abnormalities, or global dysfunction. Either/both ventricles may be affected, being right ventricular free wall more frequently involved [195]. Note, does not correlate with ECG changes or predict complications reliably (except when abnormalities seen in conjunction with ECG abnormalities). Echo here is more useful in monitoring where there is hemodynamic instability on the ICU following suspected myocardial contusion.

Where concomitant pneumothorax exists, TTE may be unhelpful and TEE required, however, beware polytrauma – where there is esophageal, maxillofacial or cervical spine injury, TEE is contra-indicated and other imaging modalities should be used. In each case, a comprehensive study should be performed, with attention to both anatomical and physiological assessment. Finally, where the hemodynamic status changes, echocardiographic studies should be repeated if the diagnosis is unclear, and may be used to guide volume replacement (section 5.2).

### 5.10 Echocardiography in shock

Shock is a global state of inadequate tissue oxygenation, is seen commonly on the ICU, and may be cardiogenic, hypovolemic, distributive, obstructive or dissociative (or any combination of the above). The use of focused ultrasound in many shock states is well-recognized [196]; however, on the ICU echocardiography may be used specifically to assist in the identification of some causes of shock, including[30-61,197]:

• Tamponade

• Cardiogenic shock

• Hypovolemia

• Massive pulmonary embolism

• Tension pneumothorax

As with all echocardiographic examinations, a comprehensive study should be performed where time allows, however, where time does not permit, the following more focused study may be considered:

• Is there pericardial fluid? – lf present, look for signs of tamponade, but note exceptions to the rule (section 5.3)

• Is the heart hypokinetic? – if so, how bad is the heart (given the level of support) which ventricle(s) is (are) involved, and are filling pressures elevated (section 5.12.2). If ischaemia is suspected, look for regional wall motion abnormalities, and signs and complications of myocardial ischaemia/infarction (section 5.1)

• Is the heart hyperkinetic? – if so, is the heart empty or volume overloaded? Look for signs of significant valvular pathology

• Is the heart "normokinetic"? – is this appropriate for the clinical state of the patient? Is it in reality relatively hypokinetic?

• How well filled is the heart? – assess IVC size in the clinical context and if distended and not collapsing with inspiration, seek potential causes (tamponade, PE etc)

• Where no cardiac indicators are seen, ultrasound of the thorax and abdomen should be considered

Where a diagnosis is suspected, a more detailed study (as discussed in the preceding sections) should be performed to confirm/refute and refine the diagnosis. This can be oriented by the dominant clinical feature [55]: systemic venous congestion (start by focusing on the right side of the heart), pulmonary venous congestion (start by focusing on the left side of the heart) or no clinical orientating signs (keep a high suspicion for hypovolemia). Finally, in addition to guiding the medical/surgical management and suggesting ongoing referral, in some cases further echocardiographic studies may be used to guide specific therapeutic interventions, i.e. to guide pericardiocentesis (look for the presence of contrast in the pericardial fluid – if necessary introduce some agitated saline – and when used, the presence of a guide wire) [198]. Other applications of ultrasound may be used to guide venous and arterial access, thoracentesis and paracentesis, however, detailed description of these techniques is outside the scope of this document which is concerned with echocardiography.

### 5.11 Failure to wean from mechanical ventilation

Weaning from mechanical ventilation fails in about 25% of patients meeting weaning criteria[199]. With discontinuation of intermittent positive pressure ventilation/continuous positive airway pressure (CPAP), increases in both LV afterload and preload are determined by withdrawal of positive end expiratory pressure (PEEP) and/or pressure support to inspiratory efforts[200,201]. In patients with LV heart disease or COPD a key or concurrent role in weaning failure can be played by a failing left heart, unable to match the increased work demand and leading to left atrial hypertension and pulmonary edema [202]. Echocardiography can thus demonstrate the cardiovascular origin of weaning failure, by detecting at end of a weaning trial [11,148]:

• A change of Doppler indexes toward an increase in left atrial pressure

• New/worsened regional wall motion abnormalities

• A decrease in LV global function

• Appearance/worsening of mitral regurgitation

Of note, and similar to the concept of stress-echocardiography, great importance has comparison of findings with a basal exam, to be done immediately before starting the weaning trial.

### 5.12 Hemodynamic measurements

Echocardiography is widely used for the hemodynamic assessment of valvular pathology – regurgitation or stenosis. Valve disease as a primary pathology leading to ICU admission is uncommon. When present, the diagnosis may be challenging to the echocardiographer as the cardiac output may be markedly reduced, or the patient hemodynamically unstable. This is particularly notable when stenosis is suspected in a low cardiac output state, as here, trans-valvular velocities may be low. Careful echocardiographic assessment of all four valves is essential in a comprehensive study, with interpretation of trans-valvular forward and regurgitant velocities being interpreted in the clinical context of the patient.

More common is the use of echocardiography for interpretation of hemodynamics in a structurally normal heart. Here, values may be obtained (using TTE or TEE) to estimate left atrial pressure, pulmonary arterial pressure and cardiac output, and guide institution and monitoring of therapeutic interventions [9,11]. It is important to remember that echocardiography cannot measure absolute pressures in any cardiac chamber, however.

#### 5.12.1 Right heart

##### Tricuspid valve (TV) disease

Disease of the tricuspid valve leading to ICU admission is extraordinarily rare.

• The commonest use of measurement across the TV in the ICU is to assess systolic Pulmonary Artery Pressure (PAPs)

• TTE assessment – generally better for hemodynamics than TEE (Parasternal inflow view, SAX at base of heart and 4 Chamber views may be used)

• TEE assessment – used where no TTE views are possible, or detailed imaging of the TV (i.e. to exclude endocarditis) is needed. Standard views include ME4Ch, modified bicaval view, transgastric short and long axis views

• Where there is free TR, color Doppler is unreliable, and PW Doppler should be performed

• Severe TR may be indicated by a regurgitant jet of short duration and low velocity

##### Pulmonary valve disease

Pulmonary valve disease rarely is an indication for ICU admission – except following cardiac surgery.

• TEE is often not helpful in assessment of the PV (particularly in the presence of aortic valve disease/replacement) and here TTE provides adequate views (PSAX)

• Where suspected, PW and CW Doppler should be performed

• In severe PR, although color Doppler may be unhelpful, PW Doppler showing regurgitation of short duration (compared with the RR interval) indicates severe PR[203]

##### Pulmonary artery pressures

This is generally calculated from the peak TR velocity[204], however:

• Where there is no TR:

- mean pulmonary artery pressure estimated from peak pulmonary artery regurgitant velocity[205].

- peak pulmonary artery systolic pressure from pulmonary acceleration time[206].

• Doppler of the pulmonary valve is dependent upon accurate placement of the cursor at the level of the valve leaflets – or pulmonary hypertension may be overestimated

• Falling PAPs may indicate failing RV rather than successful treatment of pulmonary hypertension (here the PAT may be more specific)

#### 5.12.2 Left heart

The commonest cause of valve disease in the Western World is now previous valve replacement. Elsewhere, rheumatic valve disease is still prevalent. Left-sided valvular pathology as a cause of ICU admission is more common than right-sided pathology.

##### Aortic Valve Disease

Aortic stenosis (AS) is increasingly common in the elderly. The diagnosis is suggested from calcification of the aortic valve and immobile cusps[207], visible even with limited TTE views. Of note:

• There are no agreed hemodynamics values for what constitutes significant AS in the critically ill patient

• Where LV function is poor and CO low, the measured velocity and thus derived pressure drop across the aortic valve may be correspondingly small. Peak velocity below the valve should be measured by PW Doppler across the LVOT and across valve by CW Doppler. A four-fold step-up in velocity across the aortic valve to be indicative of severe AS[208].

• AS will tend to be under-estimated in the presence of MS (as with any sequential stenotic lesions)

• ICU admission can be precipitated in patients with (known and unknown) severe congenital sub-aortic stenosis.

Aortic regurgitation (AR) is readily detectable using TTE and CW Doppler[207], even in patients on the ICU with sub-optimal echo windows. Severe AR results in high forward velocities across the aortic valve and diastolic regurgitant velocities falling to < 2 m/s at end-diastole. Where AR is catastrophic:

• Aortic velocities reach zero before end-diastole

• diastolic MR may be seen

• flow reversal in the descending aorta should be sought using PW Doppler

• M-mode of the mitral valve demonstrates premature closure.

##### Mitral valve disease

Significant mitral stenosis (MS) may mimic ARDS, presenting with poor gas exchange and bilateral pulmonary infiltrates. The history will usually suggest the underlying diagnosis. Even in the ICU, echocardiographic findings are characteristic. A precipitant for acute deterioration may be the onset of atrial fibrillation. Where the patient is admitted to the ICU in extremis, emergent balloon valvotomy or valvuloplasty may be considered. As with aortic stenosis, sequential stenotic lesions and/or a low cardiac output may lead to an underestimation of severity.

• Diagnosis of severe MS should be made using standard echocardiographic criteria[209]

• Where intervention is considered for cardiogenic shock on the ICU, TEE assessment of the valve is useful to determine suitability for valvotomy or valvuloplasty

• In extremis, a small increase in valve orifice area may improve the hemodynamics sufficiently such that the patient may be suitable for subsequent more definitive surgery

Mitral regurgitation (MR) severe enough to require ICU admission is likely to be catastrophic, with patients presenting with cardiogenic shock and/or pulmonary edema. MR of this severity may be associated with both typical and atypical echocardiographic features[210], including:

• Hyperdynamic LV (M-mode) – despite low CO state

• Reduced end-systolic volumes (2D and M-mode)

• Anatomical MV disease (best demonstrated using TEE)

• V wave cutoff sign on CW Doppler of MR trace (i.e. of short duration and low velocity)

• Color Doppler interrogation may underestimate the severity

Of note also, IPPV and drugs used for sedation alter hemodynamics sufficiently to reduce the apparent severity of MR by at least I-II grades. Thus, severe MR may only become apparent on attempting to wean from ventilator support. Where suspected, volume loading and the use of pressor agents should be considered to mimic usual (and stress) hemodynamics. In the case of suspected ischemic MR, other strategies should be employed (section 5.1 and 5.8)

##### Left atrial pressure

The left atrial pressure (LAP) may be estimated using several parameters – although widely used in the out-patient setting, the use of these techniques in the critically ill is currently not extensively validated. They may, however give a useful indication as to the presence of extremely high LAP, or a normal LAP which may be crucial in the diagnosis of many critically ill patients[147]. In addition, changes in measured indicators of LAP may suggest success (or failure) of treatment. Most measurements are easily made using TTE, even if the 2D views appear suboptimal. The critical values generally used are:

• MV deceleration time < 120 msec predicts PCWP > 20 mmHg [211]

• MV deceleration time > 153 msec predicts PCWP < 23 mmHg[212]

• Isovolumic relaxation time = 0 indicates LAP > 30 mmHg[213]

• The LAP may be estimated from the MR pressure drop, however, this is less accurate than on the right side of the heart

• Increasing E/Ea ratio > 10 indicates LAP > 15 mmHg [214]

#### 5.12.3 General measurements

##### Cardiac Output/Stroke volume

Cardiac output (CO) may be measured using many echocardiographic techniques[215,216]; however the most consistent in the critically ill uses the LVOT and aortic valve[217]

Here, the LV stroke volume is obtained by measuring the CSA of the LVOT multiplied by the trans-aortic VTI. The greatest error is in measuring the LVOT – and TEE views may well be necessary.

• TTE: LVOT diameter from PLAX view, Doppler from A4Ch view

• TEE: LVOT from MO LAX view, Doppler from TG LAX view

##### Afterload: systemic and pulmonary vascular resistance

Many echocardiographic measures may be used to estimate pulmonary vascular resistance/high pulmonary arterial pressures[218,219] including:

• A short pulmonary acceleration time (< 70–90 msec) – correlates with a high pulmonary vascular resistance[220]

• The ratio of peak tricuspid regurgitant velocity to the RVOT VTI – correlates well if the PVR is > 6.0 Wood Units[221]

• The ratio of the RV pre-ejection period to ejection time – increased with an increase in pulmonary artery diastolic pressure, pulmonary vascular resistance and mean pulmonary artery pressure[222] Of note, all parameters that are derived from the Doppler curve correlate with afterload – but this does not necessarily correlate with vascular resistance (i.e. if mean PAP is high, and PCWP is elevated, RV afterload is high but vascular resistancemay be low).

Echocardiography is not useful in the estimation of systemic vascular resistance, although esophageal Doppler (equivalent to measuring descending aortic flow using PW Doppler and 2D imaging of the aorta) is widely used as a monitoring device in the ICU to determine cardiac output and SVR.

##### Serial hemodynamic monitoring

As with any measure of hemodynamics, numbers obtained should be interpreted in the clinical context, and sequential values are generally required[12]. Prolonged insertion of the TEE probe solely to allow serial CO studies should not be considered routine, particularly where there are high right sided pressures, as gastric and esophageal pressure/heating trauma may occur. Here, the use of other means of cardiac output monitoring is probably more appropriate. If prolonged or repeated echocardiographic monitoring is required, where possible TTE should be employed to minimize esophageal trauma. Where this is not possible, minimization of the amount of US applied, together with careful probe manipulation (including leaving in a neutral position) is essential.

## Supplementary Material

Additional file 1 Minimum dataset and additional data comprising the standard adult TTE study. The table summarizes the minimum and additional data comprising the standard adult TTE study by, view, modality, structure, measurements, and derived calculations. Click here for file

Additional file 2 Minimum dataset and additional data comprising the standard adult TEE study. The table summarizes the minimum and additional data comprising the standard adult TEE study by, view, modality, structure, measurements, and derived calculations. Click here for file

Additional file 3 Proposed Levels of competence for echocardiography in ICU. The picture illustrates the proposed levels of competence for echocardiography in ICU and their relation to the accreditation process and to research and training activities. Click here for file

Additional file 4 Modular structure of the Echocardiography in ICU training programme. The picture illustrates the modular structure of the Echocardiography in ICU training programme, wich comprises emergency-introductory, basic and intermediate stages, each one with its own modules. Click here for file
