# Supplementary material for: Echocardiography practice, training and accreditation in the intensive care: document for the World Interactive Network Focused on Critical Ultrasound (WINFOCUS)
Source: Cardiovasc Ultrasound. 2008 Oct 6;6:49. doi: 10.1186/1476-7120-6-49 (PMC2586628; doi:10.1186/1476-7120-6-49)
Supplement: Additional file 1 — Minimum dataset and additional data comprising the standard adult TTE study. The table summarizes the minimum and additional data comprising the standard adult TTE study by, view, modality, structure, measurements, and derived calculations. [file 1476-7120-6-49-S1.pdf]

Table 2. Minimum dataset and additional data comprising the standard adult TTE study. *The table summarizes the minimum and additional data comprising the standard adult TTE stud by view, modality, structure, measurements, and derived calculations. Additional data are annotated with* [**A**]. *Views and measurements not supported unanimously are given in italics.* CFM = color flow mapping; PW = pulsed wave Doppler; CW = continuos wave Doppler; LV = left ventricle; RV = right ventricle; LA = left atrium; RA = right atrium; Las = LA in systoile; LVIDd/s left ventricular internal diameter in diastole and systole; LVSd/s left ventricular septal width in diastole and systole; LVPWd/s left ventricular posterior wall width in diastole and systole; MV = mitral valve; MR = mitral regurgitation; MS = mitral stenosis; LVOT = left ventricular outflow tract; AR = aortic regurgitation; AS = aortic stenosis; VSD = ventricular septal defect; IVC = inferior vena cava; SVC = superior vena cava; TV = tricuspid valve; TR = tricuspid regurgitation; Vmax, V mean = maximum and mean velocities; VTI = velocity-time integral; Pmax, P mean = maximum and mean pressure gradient; RVOT = right ventricular outflow tract; PV = pulmonary valve; PR = pulmonary regurgitation; PS = pulmonary stenosis; PA = pulmonary artery; PAPs,d = pulmonary artery pressure, systolic/diastolic; AV = aortic valve; RUPV = right upper pulmonary vein; PDA = pervium doctus arteriosum; PHT pressure half-time; DET deceleration time; IVRT = isovolumic relaxation time; RVd right ventricular cavity diameter in diastole; MVA = mitral valve area; IVS = inter-ventricular septum; SAX = short axis; Ch = chamber.

View	Modality	Structures Assessed	Measure	Calculate
**Parasternal LAX**	2D	LV cavity size, wall thickness, function	LVIDd/s, LVSd/s, LVPWd/s	FS
		RV cavity size and function		
		LA size	LAs	
		Aortic root & valve – appearance & function	annulus, root, sinuses, sino-tubular junction, ascending .aorta	
	M mode			
		LV cavity size, wall thickness	LVIDd/s, LVSd/s, LVPWd/s	FS

		RV cavity size	RV(d)	
		MV	± End Systolic separation	
		AV/aortic root/LA size	LAs, cusp separation, root	LA:Ao ratio [**A**]
	CFM			
		MV inflow / MR	AR width	AR:LVOT ratio [**A**]
		LVOT / AR		
		VSD		
**RV inflow [A]**	2D	RV cavity size and function<br>RA, IVC, SVC, ± coronary sinus<br>TV – appearance and function		
	CFM	TV inflow, TR		
	CW	TR	Vmax	PA pressure
**RV outflow**	2D	RVOT, PV, main PA	PV annulus	
	CFM	RVOT, PS, PR, PA		
	PW	RVOT	Vmax, Vmean, VTI	
	CW	PS<br>PR	Vmax, Vmean<br>Vmax PRed	Pmax, Pmean<br>PAPd
**Parasternal SAX (basal)**	2D	LA atrial septum<br>RA<br>TV – appearance and function<br>RV cavity size and function<br>PV, PA main, right<br>AV – appearance and function		
	CFM	SVC, RUPV, atrial septum [**A**]<br>TV inflow, TR [**A**]		

		RVOT, PS, PR, PA (PDA) [**A**]	Vmax, Vmean, VTI	
	PW	RVOT [**A**]		
			Vmax	PAPs
	CW	TR [**A**]	Vmax, Vmean	Pmean, Pmax
		PS [**A**]	Vmax PRed	PAPd
		PR [**A**]		
**Parasternal SAX (MV)**	2D	LV size, wall thickness, function (basal segments)		
		RV cavity size and function		
		MV – appearance and function	MVA planimetry [**A**]	
	CFM	MV inflow, MR [**A**]		
		(VSD)		
**Parasternal SAX (cords)**	2D	LV size, wall thickness, function (mid segments)		
	CFM	(VSD)		
**Parasternal SAX (apex)**	2D	LV size, wall thickness, function (apical segment)		
	CFM	(VSD)		
**Apical 4Ch**	2D	LV cavity size, wall thickness, function (IVS, lateral wall)		
		RV cavity size and function		
		LA size	Area or volume [**A**]	LA vol index[**A**]
		RA size	Area or volume [**A**]	RA vol index[**A**]
		MV – appearance and function		
		TV – appearance and function		
	CFM	MV inflow, MR		
		TV inflow, TR		
	PW	LV inflow (MV tips)	E, E DET,A	E/A ratio
			IVRT [**A**]	
	CW	MS	Vmax, Vmean [**A**]	Pmax, Pmean
				PHT [**A**] MVA

		MR<br>TS<br>TR	 Vmax, Vmean [**A**]<br>Vmax	 Pmax, Pmean<br>PAPs
**Apical 5Ch**	2D	LV cavity size, wall thickness, function [**A**]<br>LVOT, AV – appearance and function		
	CFM	LVOT, AV		
	PW	LVOT	VTI [**A**]<br>Vmax, Vmean [**A**]	SV, CO<br>Pmax, Pmean
	CW	AS<br>AR	Vmax, Vmean [**A**]<br>AR DET [**A**]	Pmax, Pmean
**Apical 2Ch**	2D	LV cavity size, wall thickness, function (anterior, inferior wall) & appearance		
**Apical LAX**	2D	LV cavity size, wall thickness, function (IVS, posterior wall) & appearance<br><br>LVOT, AV – appearance and function		
**Subcostal 4Ch**	2D<br>CFM	4 chamber structures<br>Atrial septum<br>4 chamber structures		

**Subcostal SAX**	2D	SAX structures<br>Atrial septum<br>IVC, Hepatic Veins (modified view)<br>Descending Aorta (modified view)		
	M-mode	IVC	Diameter, Respiratory variation	IVC distens. index
	CFM	SAX structures<br>Atrial septum<br>IVC , Hepatic. Veins<br>Descending. Aorta		
	PW	Hepatic Veins<br>Descending. Aorta		
**Supra-sternal**	2D	Arch		
	CFM	Arch, coarctation, PDA		
	PW	Descending. Aorta	Flow reversal	
	CW	Ascending Aorta (AS)<br>Descending. Aorta (coarctation)	Vmax, Vmean [**A**]<br>Vmax, Vmean [**A**]	Pmax, Pmean<br>Pmax, Pmean
