# Supplementary material for: Echocardiography practice, training and accreditation in the intensive care: document for the World Interactive Network Focused on Critical Ultrasound (WINFOCUS)
Source: Cardiovasc Ultrasound. 2008 Oct 6;6:49. doi: 10.1186/1476-7120-6-49 (PMC2586628; doi:10.1186/1476-7120-6-49)
Supplement: Additional file 2 — Minimum dataset and additional data comprising the standard adult TEE study. The table summarizes the minimum and additional data comprising the standard adult TEE study by, view, modality, structure, measurements, and derived calculations. [file 1476-7120-6-49-S2.pdf]

Table 3. Minimum dataset and additional data comprising the standard adult TEE study. *Table describes the minimum dataset and additional data comprising the standard adult TEE study by view, modality, structure, measurements, and derived calculations. Additional data are annotated with* [**A**]. *Views and measurements not supported unanimously are given in italics*. CFM = color flow mapping; PW = pulsed wave Doppler; CW = continuos wave Doppler; LV = left ventricle; RV = right ventricle; RWMA = regional wall motion abnormalities; LA = left atrium; RA = right atrium; Las = LA in systoile; LVIDd/s left ventricular internal diameter in diastole and systole; LVSd/s left ventricular septal width in diastole and systole; LVPWd/s left ventricular posterior wall width in diastole and systole; ESA = end systolic area; EDA = end diastolic area; FAC = fractional area change; FS = fractional shortening; MV = mitral valve; MR = mitral regurgitation; MS = mitral stenosis; LVOT = left ventricular outflow tract; AR = aortic regurgitation; AS = aortic stenosis; VSD = ventricular septal defect; IVC = inferior vena cava; SVC = superior vena cava; TV = tricuspid valve; TR = tricuspid regurgitation; Vmax, V mean = maximum and mean velocities; VTI = velocity-time integral; Pmax, P mean = maximum and mean pressure gradient; RVOT = right ventricular outflow tract; PV = pulmonary valve; PR = pulmonary regurgitation; PS = pulmonary stenosis; PA = pulmonary artery; PAPs,d = pulmonary artery pressure, systolic/diastolic; AV = aortic valve; LUPV = left upper pulmonary vein; PDA = pervium doctus arteriosum; PHT pressure half-time; DET deceleration time; IVRT = isovolumic relaxation time; RVd right ventricular cavity diameter in diastole; MVA = mitral valve area; IVS = inter-ventricular septum; IAS = interatrial septum; SAX = short axis; Ch = chamber; Sub-AS = subaortic stenosis; CO = cardiac output; SV = stroke volume.

View	Modality	Structures Assessed	Measure	Calculate
**TG-SAX (mid-papillary)**	2D	LV cavity size, wall thickness, function, RWMA<br>RV cavity size, wall thickness, function	LV EDA, LV ESA	FAC
	M mode	LV cavity size<br>IVC, Hepatic Veins	LVIDd/s, LVSd/s<br>Respiratory variation	FS<br>IVC distensibility index

**TG-SAX (basal)**	2D	MV - appearance<br>TV - appearance<br>LV: function, RWMA		
	CFM	MR/TR		
**TG-LVOT**	2D	LVOT, AV<br>MV sub-valvar apparatus		
	CFM	AR		
	PW	LVOT, AV	VTI [**A**]	SV, CO, Vmax<br>respiratory variations
	CW	LVOT, AV	Vmax, Vmean, VTI	Pmax, Pmean
**TG-RVOT**	2D	TV appearance – valve & subvalvar<br>RVOT<br>PV	 RVOT dimension<br>PV annulus [**A**]	
	CFM	TR, PR, RVOT		
	PW	RVOT/PV [**A**]<br>PR [**A**]	Vmax<br>Vmax, Pmean	PAPs
	CW	RVOTO/PV [**A**]	Vmax PRed	Vmean Pmax
**Deep TG**	2D	LV size, function<br>RV size, function<br>MV sub-valvar apparatus		
	PW	LVOT, AV	VTI, Vmax [**A**]	SV, CO, Vmax resp<br>variations
	CW	LVOT, AV<br>AR	Vmax, Vmean, VTI	Pmax, Pmean

**ME- 4Ch**	2D	LV cavity size, wall thickness, function (IVS, lateral wall)		
		RV cavity size and function		
		LA size	Area or volume [**A**]	LA vol index[**A**]
		RA size	Area or volume [**A**]	RA vol index[**A**]
		MV – appearance and function		
		TV – appearance and function		
	CFM	MV inflow, MR	Vena contracta, PISA	Severity MR
		TV inflow, TR		
	PW	LV inflow (MV tips)	E, A DET	E/A ratio
		RV inflow (TV tips)	E, A	IVRT [**A**]
		LUPV drainage	S, D, A, S VTI, D VTI,	S/D ratio, Syst Fraction
	CW	MS	Vmax, Vmean [**A**]	Pmax, Pmean
		MR	LV:LA pressure drop	PHT [**A**] MVA
		TS	Vmax, Vmean [**A**]	Pmax, Pmean
		TR	RV:RA pressure drop	Pmax,, est PAPs
**ME-commissural**	2D	LV function [**A**]		
		LA dimension	LA size	
		MV appearance, function, annular size		
	CFM	MR		
**ME-2Ch**	2D	LV function (anterior, inferior wall)		
		LAA		
		MV appearance, function		
**ME-LAX**	2D	LV function (antero-septum, posterior wall)		
		LVOT, AV – appearance and function	LVOT, annulus, Asc Aorta	
		MV appearance, function		
	CFM	MR	Vmax	Pmax,

		AS/AR<br>Sub-AS	AR width<br>Vmax , VTI	AR:LVOT [**A**]
	PW	LV inflow (MV tips)	E, A, E DET	E/A ratio
**RV inflow-outflow**	2D	TV, RV, PV		
	CFM	TR, PR./PS, RVOT obstruction		
	PW	RV inflow	E, A	
	CW	TR	RV:RA pressure drop	Pmax,, est PAPs
**AV-SAX**	2D	AV structure<br>Coronary ostia<br>IAS, PV		
	CFM	AR<br>Coronary flow<br>PR/PS		
	PW	Coronary flow	Vmax	
**Bicaval**	2D	SVC, IVC, IAS, RAA		
	M-Mode<br>CFM	SVC<br>IAS	Respiratory variations<br>Direction of shunting if PFO/ASD	SVC collapsibility. index<br>Indirect LAP
	PW	RV inflow	E, A	
	CW	TR	RV:RA pressuredrop	Pmax,, est PAPs
**UE PA**	2D	Main PA, RPA, LPA<br>Ascending Aorta	PA Diameter, Aorta diameter	
	PW	PA Doppler	VTI	CO, SV
