# Supplementary material for: Echocardiography practice, training and accreditation in the intensive care: document for the World Interactive Network Focused on Critical Ultrasound (WINFOCUS)
Source: Cardiovasc Ultrasound. 2008 Oct 6;6:49. doi: 10.1186/1476-7120-6-49 (PMC2586628; doi:10.1186/1476-7120-6-49)
Supplement: Additional file 3 — Proposed Levels of competence for echocardiography in ICU. The picture illustrates the proposed levels of competence for echocardiography in ICU and their relation to the accreditation process and to research and training activities. [file 1476-7120-6-49-S3.pdf]

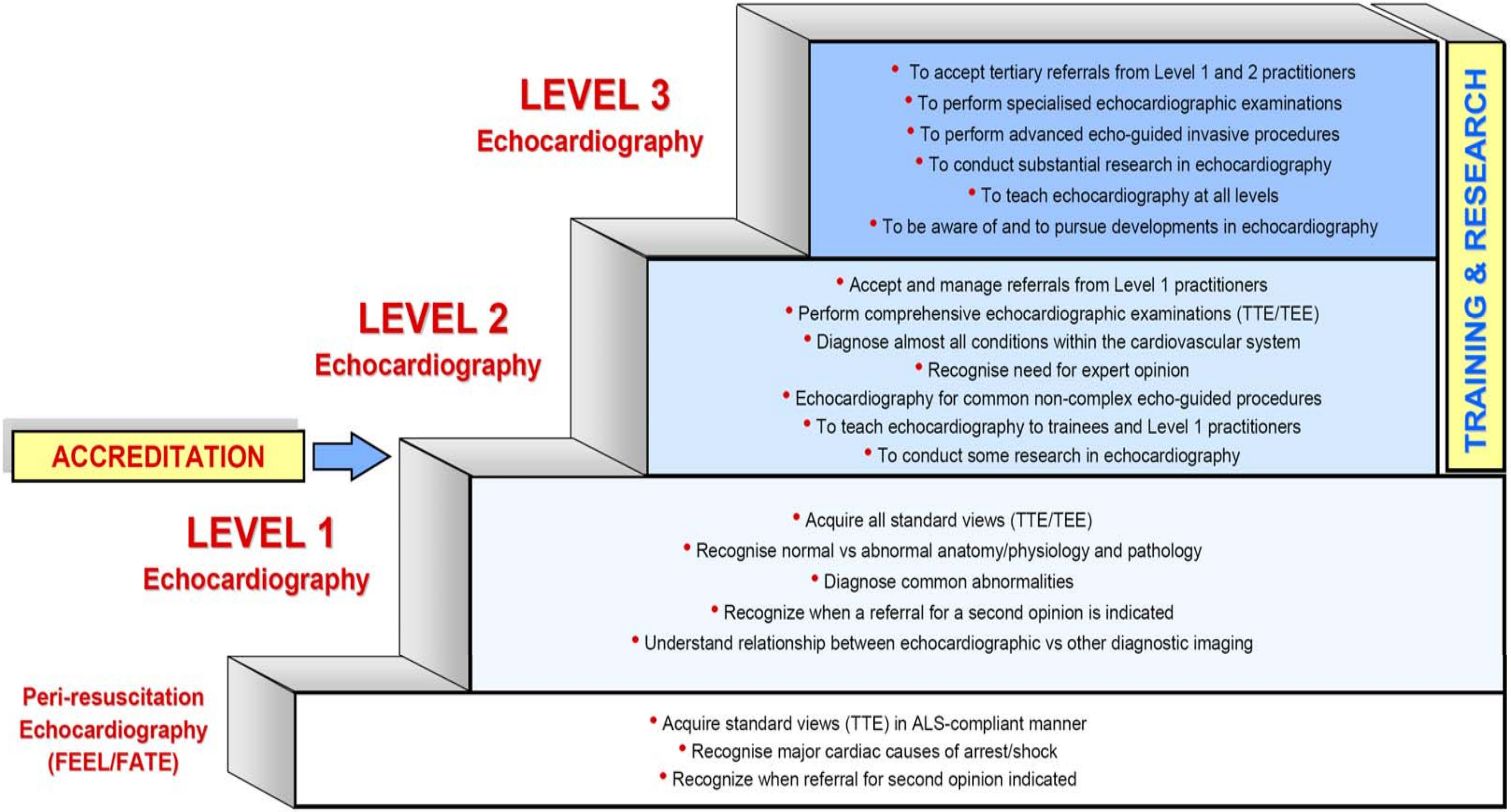

Proposed Levels of competence for echocardiography in ICU. Emergency echocardiography represents the entry level, whilst Level 1 echocardiography is achieved through a modular training programme, based on blended-learning, proctored practiced, and link to existing national/international echocardiography courses. Completion of this level would allow to undertake accreditation via a recognized examination, then giving access to Level 2 competence training and further accreditation. Cutting edge expertise is represented by Level 3 competence.
