# Supplementary material for: Echocardiography practice, training and accreditation in the intensive care: document for the World Interactive Network Focused on Critical Ultrasound (WINFOCUS)
Source: Cardiovasc Ultrasound. 2008 Oct 6;6:49. doi: 10.1186/1476-7120-6-49 (PMC2586628; doi:10.1186/1476-7120-6-49)
Supplement: Additional file 4 — Modular structure of the Echocardiography in ICU training programme. The picture illustrates the modular structure of the Echocardiography in ICU training programme, wich comprises emergency-introductory, basic and intermediate stages, each one with its own modules. [file 1476-7120-6-49-S4.pdf]

Modular structure of the Echocardiography in ICU training programme. The training programme comprises emergency-introductory, basic and intermediate stages, each one with its own modules. Theoretical and practical content of each module is delivered respectively through blended-learning and supervised practice.

MODULE TYPE	MODULES
Introductory & Emergency modules	- Principles of US<br>- Principles of Doppler<br>- "Knobology"<br>- Pitfalls and Artifacts in Echocardiography<br>- Patient safety & consent<br>- Sonoanatomy of the Heart<br>- ACLS Conformed Focused Echo (FEEL)<br>- Screening Focused Echo (FATE)
Basic modules	- Qualitative evaluation of the Left ventricle (LV)<br>- Qualitative evaluation of the Right ventricle (RV)<br>- Measuring Inferior (IVC) &/or Superior (SVC) Vena Cava diameter<br>- Measurement of LV Outflow Tract (LVOT) peak velocity and VTI<br>- Masurement of Cardiac Output (CO)<br>- Estimation of peak Pulmonary Artery Pressure (PAPs)
Intermediate modules: Assessment of Ventricular function	- Quantification of LV systolic function<br>- Quantification of RV systolic function<br>- LV diastolic function<br>- RV diastolic function<br>- Estimation of Left Atrial Pressure (LAP)<br>- Detection of Myocardial ischemia
Intermediate modules: Valvular Disease	- Aortic Stenosis (AS)<br>- Aortic Regurgitation (AR)<br>- Mitral Regurgitation (MR)<br>- Mitral Stenosis (MS)<br>- Tricuspid valve (TV)<br>- Pulmonary valve (PV)
Intermediate modules: Clinical scenarios	- General Approach to the Shocked patient<br>- Hypovolaemia, Volume Responsiveness & Filling Status<br>- Tamponade<br>- The Sepsis Syndromes<br>- Effects of Preload and Aferload<br>- Hypoxaemia<br>- Anatomical shunt<br>- Acute Cor Pulmonale (Pulmonary Embolism, ARDS)<br>- Complications of Acute Myocardial Infarction<br>- Chest trauma<br>- Failure to wean from Mechanical Ventilation<br>- Hemodynamic Measurements
